# Challenges in Collating Spirometry Reference Data for South-Asian Children: An Observational Study

**DOI:** 10.1371/journal.pone.0154336

**Published:** 2016-04-27

**Authors:** Sooky Lum, Vassiliki Bountziouka, Philip Quanjer, Samatha Sonnappa, Angela Wade, Caroline Beardsmore, Sunil K. Chhabra, Rajesh K. Chudasama, Derek G. Cook, Seeromanie Harding, Claudia E. Kuehni, K. V. V. Prasad, Peter H. Whincup, Simon Lee, Janet Stocks

**Affiliations:** 1 Respiratory, Critical Care & Anaesthesia section (Portex Unit), UCL, Institute of Child Health, London, United Kingdom; 2 Department of Pulmonary Diseases and Department of Paediatrics-Pulmonary Diseases, Erasmus Medical Centre, Erasmus University, Rotterdam, Netherlands; 3 Department of Paediatric Pulmonology, Rainbow Children’s Hospital, Bangalore, India; 4 Clinical Epidemiology, Nutrition and Biostatistics section, UCL, Institute of Child Health, London, United Kingdom; 5 Institute for Lung Health, NIHR Leicester Respiratory Biomedical Research Unit, and Department of Infection, Immunity & Inflammation, University of Leicester, Leicester, United Kingdom; 6 Department of Pulmonary Medicine, Vallabhbhai Patel Chest Institute, University of Delhi, Delhi, India; 7 Community Medicine Department, PDU Medical College, Rajkot, Gujarat, India; 8 Population Health Research Institute, St George’s, University of London, London, United Kingdom; 9 Diabetes & Nutritional Sciences Division, Kings College London, London, United Kingdom; 10 Institute of Social and Preventative Medicine, University of Bern, Switzerland; 11 Department of Physiology, Vemana Yoga Research Institute, Hyderabad, India; University Children's Hospital Bern, SWITZERLAND

## Abstract

Availability of sophisticated statistical modelling for developing robust reference equations has improved interpretation of lung function results. In 2012, the Global Lung function Initiative(GLI) published the first global all-age, multi-ethnic reference equations for spirometry but these lacked equations for those originating from the Indian subcontinent (South-Asians). The aims of this study were to assess the extent to which existing GLI-ethnic adjustments might fit South-Asian paediatric spirometry data, assess any similarities and discrepancies between South-Asian datasets and explore the feasibility of deriving a suitable South-Asian GLI-adjustment.

**Methods:**

Spirometry datasets from South-Asian children were collated from four centres in India and five within the UK. Records with transcription errors, missing values for height or spirometry, and implausible values were excluded(n = 110).

**Results:**

Following exclusions, cross-sectional data were available from 8,124 children (56.3% male; 5–17 years). When compared with GLI-predicted values from White Europeans, forced expired volume in 1s (FEV_1_) and forced vital capacity (FVC) in South-Asian children were on average 15% lower, ranging from 4–19% between centres. By contrast, proportional reductions in FEV_1_ and FVC within all but two datasets meant that the FEV_1_/FVC ratio remained independent of ethnicity. The ‘GLI-Other’ equation fitted data from North India reasonably well while ‘GLI-Black’ equations provided a better approximation for South-Asian data than the ‘GLI-White’ equation. However, marked discrepancies in the mean lung function z-scores between centres especially when examined according to socio-economic conditions precluded derivation of a single South-Asian GLI-adjustment.

**Conclusion:**

Until improved and more robust prediction equations can be derived, we recommend the use of ‘GLI-Black’ equations for interpreting most South-Asian data, although ‘GLI-Other’ may be more appropriate for North Indian data. Prospective data collection using standardised protocols to explore potential sources of variation due to socio-economic circumstances, secular changes in growth/predictors of lung function and ethnicities within the South-Asian classification are urgently required.

## Introduction

Lung function tests are an integral part of clinical management of respiratory disease but reliable interpretation of results relies on availability of suitable reference data to help distinguish the effects of disease from those of growth and development. Appropriate reference equations are therefore crucial both in clinical management and for interpretation of clinical trials in which lung function is a primary outcome[1,2]. In addition to the major determinants of height, age and sex, lung function is also influenced by ethnicity.[3,4] Some studies have suggested that these differences may be primarily attributed to social deprivation[5,6]. However, while there is evidence that severe deprivation may impact negatively on both growth and lung function,[7,8] recent studies in developed countries have shown that the contribution of socio-economic factors is minimal[9–13] and that ethnic differences in lung function persist even when such factors are taken into account.

Since publication of the most recent ERS/ATS guidelines for spirometry in 2005 [14] there have only been two publications on spirometry reference ranges for Indian adults.[15,16] By contrast, of the various publications reporting spirometry reference equations for children from the Indian sub-continent (hereafter referred to as South-Asian), seven have been published in the past 15 years. [8,17–22] These have, however, been derived using simple regression techniques based on data collected in different parts of the Indian subcontinent, using different equipment in children of different age ranges and socio-economic backgrounds and may therefore not be generalisable.

Development of sophisticated statistical modelling techniques for deriving more robust reference equations has provided opportunities to improve interpretation of lung function results across the age span, with the GLI publishing the first global all-age, multi-ethnic reference equations for spirometry in 2012[3]. These equations were available for 5 distinct ethnic groups, i.e. Caucasian (hereafter referred to as “White”), African-American (hereafter referred to as “Black”), North-East Asian (e.g. North China, Korea), South-East Asian (e.g. South China, Thailand, Taiwan) and Other (consisting of groups other than the 4 main groups and those of mixed ethnic origin). Although some spirometry data South-Asians were provided to the GLI team, results from these studies were sufficiently disparate with respect both to mean results and their distribution to preclude the necessary combination of datasets to derive reliable reference equations [3]. The lack of a ‘normal range’ for South-Asian children currently limits the application and interpretation of their lung function tests.

Two recent studies of 5–12 year old South-Asian school children in London, UK and in Bangalore, India using identical equipment, protocol and quality control(QC)[7,23] showed that after adjusting for height, age and sex[3], average forced expired volume in 1 second (FEV_1_) and forced vital capacity (FVC) were very similar in Indian urban children residing in Bangalore city to those living in the UK; both being approximately 11% (~0.9z-score) lower than that predicted for White European children[7], with no ethnic differences in the FEV_1_/FVC ratio. The similarity of results from these two studies, suggested that it should be possible to derive an additional GLI-adjustment (coefficient) suitable for use in South-Asian children. The primary aim of this study was to assess the extent to which existing GLI-ethnic adjustments might fit South-Asian paediatric spirometry data. The secondary aim was to assess any similarities and discrepancies between South-Asian datasets and the feasibility of deriving a suitable GLI-adjustment if needed for interpreting such data. Some results from this study have been reported in abstract form[24].

## Materials and Methods

UK centres who had recently (last 15 years) published or collected spirometry data from healthy South-Asian subjects aged 5–18 years or Indian centres who had either submitted such data to the GLI team or recently published their findings were invited to collaborate. See supporting information in S1 File, Section 1.1 for recruitment and exclusion criteria and S1 Table for further details. All centres indicating willingness to participate were requested to provide information on population characteristics (e.g. age, sex, anthropometry, ethnicity, socio-economic circumstances (SEC)) and equipment used. Data were anonymised before submission to this collaboration and came from research studies for which full local ethics approvals were obtained, i.e. from the M.S. Ramaiah Medical College and Teaching Hospitals Ethics Board, Bangalore; Vallabhbhai Patel Chest Institutional Ethics Committee, Delhi; Ethical Committee of the Government Medical College, Surat, Gujarat; Ethical Committee of the National Institute of Nutrition, Hyderabad; Multicentre Research Ethics Committee, UK (for CHASE and DASH study); Leicestershire Research Ethics Committee (for the two Leicester studies) and Research Ethics Committee: London-Hampstead: REC 10/H0720/53 (SLIC study). Parental written consent and verbal assent from each child were obtained prior to assessments.

### Data

Among the 11 centres (six UK and five Indian centres) contacted, ten centres responded positively although only nine were able to submit the requested data within the available time frame. Datasets (n = 8413 initially) were available from four centres in India (Bangalore, Delhi, Hyderabad and Gujarat) [7,17,18,21,25] and five in the UK (three in London and two in Leicester; Table 1)[10,11,23,26–28]. Records with transcription errors that could not be resolved, with missing values for height, FEV_1_ or FVC, or where lung function values were deemed implausible (i.e. FEV_1_/FVC ratio >1.0; FEV_1_ or FVC ≤0.3L) were discarded (n = 110).

**Table 1:** Summary of studies included in the collation of South-Asian data.

Centre	Publication (author, year)	Region where data collection performed	Date of collection	Number of healthy subjects	Ethnicity (based on ancestral origin#)	Age range (year)	Birth data (Y/N)	Sitting height (Y/N)	SEC	Spirometer used	Data available for QC? (Y/N)
A	Sonnappa, 2015[7]	Bangalore, India	2013	782	100% Indian	5.0–16.4	N	Y	Y	Easy-on-PC, ndd	Y
B	Chhabra, 2012[17]	Delhi, India	2007–10	670	100% Indian	6–17	N	N	N	Medisoft Micro 5000	Y
C	Doctor TH, 2010[18]	South Gujarat, India	2007–08	648	100% Indian	8.0–13.9	N	N	N	Spirolab II, MIR 010	N
D	Raju, 2003[25]; Raju, 2004[21]	Hyderabad, India	1995–97	2540	100% Indian	5–15	N	Y	Y	Vitalograph	N
E	Barone-Adesi, 2015[28]	London, Birmingham, Leicester, UK (CHASE study)	2004–07	1547	32% Indian; 23% Bangladeshi; 37% Pakistani; 8% SA Other	9.0–11.1	N	Y	Y	Vitalograph compact 2	N
F	Whitrow, 2008[11]	London, UK (DASH study)	2001–02	1064	46% Indian; 18% Bangladeshi; 36% Pakistani	11.2–13.9	N	Y	Y	Micro Plus, MicroMedical	N
G	Whittaker, 2005[26]	Leicester city, UK	2001–02	177	Not specified	6.5–11.5	Y	Y	Y	Jaeger Masterscope	N
H	Stripolli, 2013[10]	Leicester, UK (LRC)	2006–10	210	Not specified	8.6–14.1	Y	N	Y	Pneumotrac, Vitalograph	N
I	Lum, 2015[9]	London, UK (SLIC study)	2011–13	486	68% Indian; 11% Bangladeshi; 8% Pakistani; 13% Sri Lankan /mixed SA	5.3–11.5	Y	Y	Y	Easy-on-PC, ndd	Y

Abbreviations: SA: South-Asian; SEC: Socio-economic circumstances; Y: Yes; N: No; QC: Quality control–Y indicates that data were readily available for independent inspection and over-read of flow-volume curves; SLIC: “Size and Lung function In Children”; CHASE: “Child Heart And health Study in England”; DASH: “Determinants of Adolescent Social wellbeing and Health”; LRC: Leicester Respiratory Cohort”. # Ancestral origin determined via parental questionnaire.

### Data management and statistical analysis

Results are presented as mean(SD) for continuous variables or as frequencies(%) for categorical variables. Anthropometry was expressed as sex-specific z-scores based on Indian growth charts derived from well-nourished children[29]. Only three studies were able to provide spirometric flow-volume curves for retrospective quality check on data provided (Table 1). To ascertain overall distribution of data from each study, FEV_1_, FVC, and FEV_1_/FVC were initially expressed as z-scores using the GLI-2012 equations for White subjects as a baseline[3].

Within each centre, lung function results were then inspected to ascertain i) the relationship between the lung function z-scores against age and height to ensure that there were no trends in residuals (see S1 File, section 1.2 for details)[3] ii) the spread of data between-subjects iii) whether offsets in FEV_1_ and FVC relative to the ‘GLI-White’ equation were proportional, as had been observed in over 60 datasets previously submitted to the GLI-2012[30]. After assessing the South-Asian spirometry data in relation to White subjects, the exercise was repeated to assess the fit of existing GLI-ethnic adjustments [3]. The appropriateness of any given reference equation to specific datasets was also ascertained by checking the percentage of healthy subjects within each centre with results falling at or below the 5^th^ centile (i.e. 5% lower limit of normal (LLN) ≤ -1.645 z-scores).

Similarities and discrepancies between datasets, including the potential impact of SEC in Indian-based studies, were examined. Prior to deriving any new South-Asian GLI-adjustment factors (S1 File, Section 1.2.1), datasets with non-proportional reductions in FEV_1_ and FVC, were excluded and the remaining data were analysed both before and after removing additional datasets that were visibly discrepant from others with respect to either mean offset or between-subject variability.

Analyses were performed using IBM SPSS Statistics version 22 (IBM Corp. Armonk, NY). One-way ANOVA was performed to compare the lung function indices (z-scores based on GLI-White) between centres. Post-hoc pairwise comparisons were subsequently performed using the Tukey’s honestly significant difference test which takes into account sample size when calculating the 95% Confidence Intervals (CI).

## Results

Data for analysis were available from 8,124 children (56.3% male; age range 5–17 years), the majority of whom were of Indian origin (Tables 1 and 2).

**Table 2:** Group characteristics and spirometry results (based on GLI-White equations) according to centre.

Centre	A	B	C	D	E	F	G	H	I	Total
Country	India (Bangalore)	India(Delhi)	India(Gujarat)	India(Hyderabad)	UK(CHASE)	UK(DASH)	UK(Leicester)	UK(LRC)	UK(SLIC)	
Subjects, n	782	670	648	2540	1547	1064	177	210	486	8124
Boys (%)	57%	55%	62%	61%	49%	61%	40%	52%	48%	56%
Age (y)	9.9(2.2)	11.6(3.3)	10.7(1.3)	10.0(3.1)	9.9 (0.4)	12.6(0.6)	9.0 (1.4)	11.8(1.1)	8.3(1.6)	10.4(2.5)
zHeight#[29]	-0.60(1.15)	0.49(1.02)	0.13(1.21)	-0.63(1.00)	0.18(1.02)	0.20(1.03)	0.18(1.02)	0.20 (1.00)	0.31(0.99)	-0.11 (1.13)
zWeight#[29]	-0.62(1.18)	0.29(0.97)	-0.06(1.06)	-0.97(0.82)	0.27(1.05)	0.23(1.01)	0.19(1.01)	0.24(1.07)	0.16(0.97)	-0.24(1.13)
zFEV_1_ (GLI-W)	-1.26(0.90)	-0.67(0.87)	-1.57(0.90)	-1.56(1.08)	-1.15(1.18)	-1.21(1.04)	-0.37(0.94)	-1.13(1.04)	-0.91(0.86)	-1.26(1.08)
FEV_1_ (%pred, GLI-W)	85.0 (10.7)	92.1 (10.1)	81.6 (10.6)	81.0 (13.5)	86.6 (13.8)	85.7 (12.3)	95.7 (11.1)	86.8 (12.3)	89.0(10.4)	85.0 (13.0)
zFVC (GLI-W)	-1.19(0.95)	-0.54(0.90)	-1.65(0.97)	-1.96(1.08)	-1.13(1.24)	-1.07(1.61)	-0.67(0.93)	-1.28(1.00)	-0.81(0.86)	-1.36(1.24)
FVC (% pred, GLI-W)	86.0 (11.1)	93.7 (10.7)	80.9 (11.3)	76.7 (13.0)	87.0 (14.3)	87.8 (18.9)	92.1 (11.1)	85.2 (11.4)	90.3 (10.4)	84.1 (14.7)
zFEV_1_/FVC (GLI-W)	-0.14(0.86)	-0.23(0.95)	0.15(0.86)	0.98(0.98)	0.06(1.28)	0.12(1.63)	0.66(0.98)	0.29(1.04)	-0.22(0.92)	0.32 (1.22)
Proportion of children with lung function ≤-1.64 z-scores (i.e. ≤5^th^ centile) according to the GLI-White equations, n (%)^a^										
zFEV_1_(GLI-W)	36.2%	13.3%	47.5%	45.9%	32.1%	31.5%	6.2%	30.5%	19.8%	35.0%
zFVC (GLI-W)	31.3%	10.6%	51.2%	61.1%	32.2%	38.2%	13.6%	32.9%	16.0%	40.3%
zFEV_1_/FVC (GLI-W)	3.2%	5.4%	2.9%	1.2%	9.1%	14.6%	1.1%	3.8%	6.6%	5.5%

Data presented as Mean(SD) unless otherwise specified. #According to Khadilkar growth reference; Abbreviation: z: z-score (i.e. standard deviation score) %pred: percent predicted; GLI-W: GLI reference equations based on White European subjects.

^a^If the reference equations are appropriate, 5% of a healthy population would be expected to fall at or below the 5^th^ centile (lower limit of normal).

### Anthropometry

Based on the Indian growth charts[29], after adjusting for sex and age, South-Asian children residing in the UK were significantly taller and heavier (by 0.56z-scores and 0.84z-scores respectively) than those living in India (S2 Table). While anthropometry was similar between UK centres, mean values from Indian centres ranged from -0.63 to +0.49z-scores for height and -0.97 to +0.29z-scores for weight (Table 2), children from Northern India (Delhi-centre B) being tallest and heaviest.

### Spirometry

**Fig 1:**
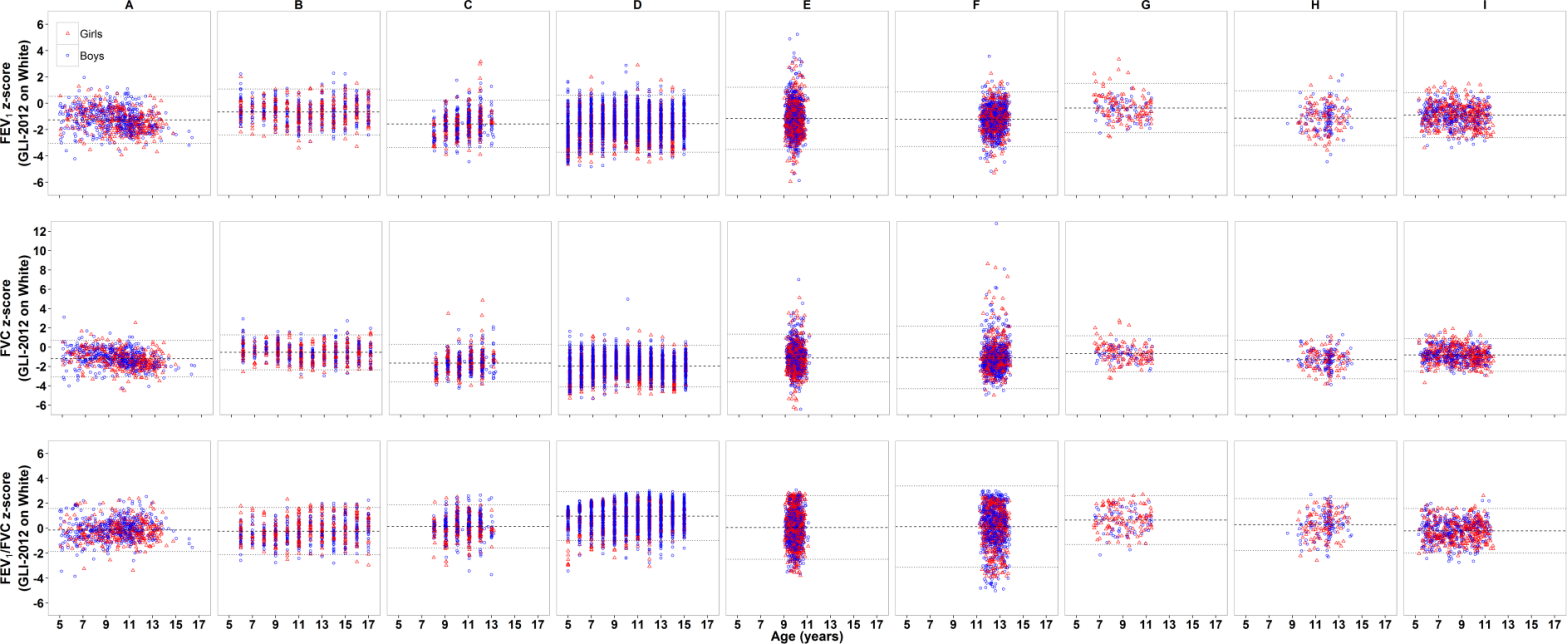
Distribution of lung function data (based on GLI-White) vs. age according to centre. *Symbols*: *Blue denotes data from boys and Red denotes data from girls*. *Dashed line = mean value and dotted lines 95% limits of agreement (Mean ± 2 SD) for each dataset*. Centres: A = Bangalore; B = Delhi; C = Gujarat; D = Hyderabad; E = CHASE (London); F = DASH (London); G = Leicester City; H = LRC (Leicester); I = SLIC (London). Note the different scales used on the y-axis reflecting the greater spread of FVC than FEV_1_ data. Note: Age was only recorded to the nearest year for data from Centres B, C and D.

Lung function results are summarised in Table 2. When compared with GLI-predicted values from White Europeans, FEV_1_ in South-Asian children were on average 15% lower, ranging from 4–19% (-0.37 to -1.57 z-scores) according to centre, with an excessive proportion of healthy children (i.e. >>5%) falling below the lower limit of normal (LLN) as defined by the 5^th^ centile. Similar results were found for FVC (average 16% lower; range 6–23%), with Centre B (Delhi) and Centre G (Leicester) having significantly larger FEV_1_ and FVC than those from all other centres (p<0.01) except Centre I, in whom zFVC was similar to that in Centre G (p = 0.93). However, proportional reductions in FEV_1_ and FVC within all but two datasets (D and G) meant that FEV_1_/FVC generally remained independent of ethnicity[3]. The distributions of z-scores according to centre are shown in the Fig 1, illustrating the wide scatter of data from some centres. When data were limited to only those from India (i.e. excluding Pakistani, Bangladeshi, Sri Lankan and Other/mixed South-Asian), results were similar to those observed from all data.

### Associations between extreme poverty and lung function

Among the UK studies, lung function differences were not explained by socio-economic factors[9–11]. By contrast, the association between SEC and lung function among children living in India is clearly illustrated in data from Bangalore(A) where both anthropometry and lung volumes were significantly lower in those living in rural areas or exposed to poorer SEC (Table 3)[7]. Anthropometry and lung function were also higher in those from high SEC compared to those from medium or low SEC in the Hyderabad(D) dataset, although the distinction between the latter groups was less clear. Details of SEC were not available from the remaining two India-based studies (B and C).

**Table 3:** Association between extreme poverty and lung function in children residing in India.

Centre	A_1_	A_2_	A_3_	D_1_	D_2_	D_3_
Country	Bangalore(urban)	Bangalore(semi-urban)	Bangalore(rural)	Hyderabad(high SEC)	Hyderabad (medium SEC)	Hyderabad(low SEC)
Subjects, n	383	234	165	1002	1018	529
Boys (%)	68%	43%	50%	50%	52%	100%
Age (y)	9.0 (1.9)	11.3 (2.0)	10.0 (2.0)	10.2 (3.1)	9.8 (3.0)	10.0 (3.2)
zHeight#	0.06 (0.90)	-1.19 (1.00)	-1.30 (0.98)	-0.29 (0.92)	-0.83 (0.93)	-0.92 (1.07)
zWeight#	0.13 (0.93)	-1.28(0.92)	-1.43 (0.91)	-0.65 (0.80)	-1.13 (0.77)	-1.29 (0.75)
zFEV_1_ (GLI-W)	-0.93 (0.85)	-1.46 (0.77)	-1.75 (0.88)	-1.30 (1.05)	-1.76 (1.09)	-1.69 (1.08)
zFVC (GLI-W)	-0.86 (0.86)	-1.40 (0.79)	-1.67 (1.05)	-1.73 (1.02)	-2.14 (1.12)	-2.09 (1.08)
zFEV_1_/FVC(GLI-W)	-0.16 (0.80)	-0.19 (0.75)	-0.06 (1.10)	1.02 (0.93)	0.94 (1.02)	0.95 (0.98)

Data presented as Mean(SD) unless otherwise specified; #According to Khadilkar growth reference[29]; Abbreviation: SEC: socio-economic circumstance; GLI-W: according to GLI-White equations[3]. Although confident of the FEV_1_ data, authors of the Hyderabad study suspected that the relatively low and non-proportional change in FVC and hence the elevated FEV_1_/FVC may have been due to difficulties in children achieving a full forced expiration in this field study.

### Extent to which existing GLI-ethnic adjustments might fit South-Asian data

The extent to which the existing GLI-South-East Asian equations (derived from subjects from South China, Thailand and Taiwan, who are geographically closest to those originating from the Indian subcontinent) fitted the South-Asian data is summarised in Table 4. While relative offsets for zFEV_1_ and zFVC were smaller than when compared with GLI-White equations, the GLI-South-East Asian equations provided a poorer fit for zFEV_1_/FVC, and an excessive proportion of healthy children had results that fell below the 5^th^ centile for all outcomes. While the entire South-Asian dataset fitted the predicted values for Black-African origin subjects (15% lower than White subjects) reasonably well (Table 5), marked differences in relative mean offsets and distribution of results (SD of z-scores >>1.0) between the various South-Asian datasets were still observed.

**Table 4:** Spirometry data from South-Asian subjects according to GLI-South-East Asian reference.

Centre	A	B	C	D	E	F	G	H	I	Total
n	782	670	648	2540	1547	1064	177	210	486	8124
zFEV_1_ (GLI-SEA)	-0.47(0.95)	0.17(0.93)	-0.80(0.97)	-0.81(1.15)	-0.31(1.26)	-0.42(1.12)	0.53(1.02)	-0.30(1.12)	-0.09(0.93)	-0.46(1.15)
zFVC (GLI-SEA)	-0.16(1.02)	0.56(0.97)	-0.65(1.07)	-1.02(1.16)	-0.05(1.34)	-0.01(1.76)	0.44(1.02)	-0.22(1.08)	0.26(0.94)	-0.33(1.35)
zFEV_1_/FVC (GLI-SEA)	-0.64(0.90)	-0.72(1.00)	-0.33(0.91)	0.53(1.02)	-0.43(1.33)	-0.35(1.72)	0.18(1.01)	-0.18(1.10)	-0.72(0.95)	-0.15(1.28)
***Proportion of children with lung function below the 5***^***th***^ ***centile according to the GLI-South-East Asian equations***^a^ ***(%)***										
zFEV_1_ (GLI-SEA)	10.5%	2.1%	17.3%	22.6%	12.1%	13.3%	1.1%	10.0%	3.9%	14.2%
zFVC (GLI-SEA)	6.3%	0.6%	16.2%	28.2%	8.1%	11.2%	1.1%	8.6%	1.2%	14.1%
zFEV_1_/FVC (GLI-SEA)	10.7%	17.6%	7.3%	3.0%	17.2%	19.7%	5.1%	10.5%	17.5%	11.3%

Data presented as Mean (SD) unless otherwise specified. Centres A = Bangalore; B = Delhi; C = Gujarat; D = Hyderabad; E = CHASE (London); F = DASH (London); G = Leicester City; H = LRC (Leicester); I = SLIC (London)

^a^If the reference equations are appropriate, 5% of a healthy population would be expected to fall at or below the 5^th^ centile (LLN). When data from Centres D and G were excluded (non-proportional reduction in FEV_1_ and FVC), group mean(SD) for zFEV_1_ was -0.33(1.12) z-scores; zFVC: -0.03(1.32) and zFEV_1_/FVC: -0.48(1.26), while proportion of children with lung function below the 5^th^ centile according to GLI-SE Asian equations were 10.7%, 7.9% and 15.4% respectively.

**Table 5:** Spirometry data from South-Asian subjects according to GLI-Black reference.

Centre	A	B	C	D	E	F	G	H	I	Total
n	782	670	648	2540	1547	1064	177	210	486	8124
zFEV_1_ (GLI-B)	-0.06 (0.96)	0.57 (0.93)	-0.36 (0.96)	-0.39(1.17)	0.09 (1.27)	0.01 (1.11)	0.89 (1.0)	0.10 (1.11)	0.29 (0.92)	-0.05(1.16)
zFVC (GLI-B)	0.08 (1.00)	0.78 (0.97)	-0.38 (1.04)	-0.75 (1.17)	0.17 (1.33)	0.25 (1.72)	0.61 (0.97)	0.01 (1.06)	0.46 (0.91)	-0.08(1.33)
zFEV_1_/FVC (GLI-B)	-0.25 (0.87)	-0.34 (0.96)	0.04 (0.87)	0.88 (0.99)	-0.05 (1.29)	0.02 (1.65)	0.56 (0.99)	0.19 (1.06)	-0.33 (0.92)	0.22(1.23)
***Proportion of children with lung function below the 5***^***th***^ ***centile according to the GLI-Black equations***^a^ ***(%)***										
zFEV_1_ (GLI-B)	4.0%	0.7%	7.4%	13.8%	6.0%	6.7%	0	5.2%	1.1%	7.6%
zFVC (GLI-B)	3.7%	0.3%	9.4%	21.4%	5.9%	7.3%	1.1%	6.2%	0.4%	10.1%
zFEV_1_/FVC (GLI-B)	3.8%	7.5%	3.5%	1.5%	10.9%	15.2%	1.1%	4.3%	7.8%	6.4%

Data presented as Mean (SD) unless otherwise specified. Centres A = Bangalore; B = Delhi; C = Gujarat; D = Hyderabad; E = CHASE (London); F = DASH (London); G = Leicester City; H = LRC (Leicester); I = SLIC (London)

^a^If the reference equations are appropriate, 5% of a healthy population would be expected to fall at or below the 5^th^ centile (LLN). When data from Centres D and G were excluded (non-proportional reduction in FEV_1_ and FVC), group mean(SD) for zFEV_1_ was 0.08 (1.11) z-scores; zFVC: 0.20 (1.30) and zFEV_1_/FVC: -0.11 (1.21), while proportion of children with lung function below the 5^th^ centile according to GLI-Black equations were 4.9%, 5.1% and 8.9% respectively.

Although not appropriate for most of the centres, application of the ‘GLI-Other’ reference appeared to fit data from Centre B (northern South-Asian) relatively well, with mean (SD) for both FEV_1_ and FVC approximating 0 (1), albeit with a slight negative offset for FEV_1_/FVC (Table 6). Under such circumstances, use of an adjusted LLN to reflect the actual 5^th^ centile for FEV_1_/FVC in that dataset (Table 7; see also S1 File, section 2.1.1) could avoid over-diagnosing abnormalities. Similarly, although the ‘Other’ equation fitted FVC data from centre G, non-proportional differences in FEV_1_ and FVC meant that there would be considerable under-estimation of airway disease unless an adjusted LLN to reflect the 5^th^ centile was applied to data from this centre (Table 7).

**Table 6:** Spirometry data from South-Asian subjects according to GLI-Other reference.

Centre	A	B	C	D	E	F	G	H	I	Total
n	782	670	648	2540	1547	1064	177	210	486	8124
zFEV_1_ (GLI-O)	-0.73(0.96)	-0.10(0.93)	-1.05(0.96)	-1.05(1.16)	-0.59(1.27)	-0.67(1.11)	0.23(1.00)	-0.58(1.11)	-0.36(0.92)	-0.72(1.15)
zFVC (GLI-O)	-0.59(1.06)	0.15(1.02)	-1.10(1.10)	-1.47(1.23)	-0.50(1.40)	-0.44(1.83)	-0.01(1.04)	-0.68(1.13)	-0.17(0.97)	-0.77(1.41)
zFEV_1_/FVC (GLI-O)	-0.33(0.90)	-0.42(1.00)	-0.02(0.91)	0.85(1.03)	-0.12(1.34)	-0.04(1.72)	0.50(1.02)	0.13(1.10)	-0.41(0.96)	0.16(1.28)
***Proportion of children with lung function below the 5***^***th***^ ***centile according to the GLI-Other equations***^a^ ***(%)***										
zFEV_1_ (GLI-O)	17.0%	3.9%	24.8%	29.6%	18.7%	17.7%	1.7%	15.2%	9.1%	20.1%
zFVC (GLI-O)	13.6%	2.7%	29.8%	43.0%	17.8%	20.7%	4.5%	20.5%	5.3%	24.4%
zFEV_1_/FVC (GLI-O)	5.9%	9.7%	4.3%	1.9%	12.3%	16.3%	1.7%	5.2%	11.3%	7.6%

Data presented as Mean (SD) unless otherwise specified. Centres A = Bangalore; B = Delhi; C = Gujarat; D = Hyderabad; E = CHASE (London); F = DASH (London); G = Leicester City; H = LRC (Leicester); I = SLIC (London)

^a^If the reference equations are appropriate, 5% of a healthy population would be expected to fall at or below the 5^th^ centile (LLN). When data from Centres D and G were excluded (non-proportional reduction in FEV_1_ and FVC), group mean(SD) for zFEV_1_ was -0.60(1.11) z-scores; zFVC: -0.47(1.38) and zFEV_1_/FVC: -0.18(1.26), while proportion of children with lung function below the 5^th^ centile according to GLI-Black equations were 16.2%, 16.3% and 10.5% respectively.

**Table 7:** Spirometry data from Delhi (Centre B) and Leicester (Centre G) according to GLI-Other reference.

Centre	N	zFEV_1_	zFVC	zFEV_1_/FVC	%≤LLN zFEV_1_	%≤LLN zFVC	% ≤LLN zFEV_1_/FVC	Adj LLN^ǂ^ zFEV_1_	Adj LLN^ǂ^ zFVC	Adj LLN^ǂ^ zFEV_1_/FVC
B	670	-0.10(0.93)	0.15(1.02)	-0.42(1.00)	3.9%	2.7%	9.7%	-1.55	-1.48	-1.96
G	177	0.23(1.00)	-0.01(1.04)	0.50(1.02)	1.7%	4.5%	1.7%	-1.27	-1.60	-1.34

Data presented as Mean (SD) unless otherwise specified. Abbreviations: GLI-O: GLI-“Other” reference; LLN: Lower limit of normal (equates to ≤ -1.645 z-scores). If the reference equations are appropriate, 5% of a healthy population would be expected to fall at or below the 5^th^ centile (LLN); Adj LLN: LLN adjusted for the actual 5^th^ centile according to centre.

This exercise was not repeated for the GLI-North-East Asian prediction equations since on average these are only 3% lower than GLI-White, and would therefore under-estimate lung function for all South-Asian data.

### Derivation of new GLI-adjustments for South-Asian children

The marked discrepancies in the mean lung function z-scores between centres (Table 2) especially when examined according to SEC (Table 3), meant that not only did none of the existing GLI-equations fit data from all of the individual centres, but precluded derivation of a single South-Asian GLI-adjustment. As data from Centres D and G had non-proportional differences in FEV_1_ and FVC (Table 2; Tables 4–6), these data were excluded from subsequent attempts to derive new South-Asian GLI-adjustments. Data with similar mean offsets were collated before modelling the data as described in the S1 File. The selection of datasets for inclusion within different models were based on the i) significantly higher anthropometric (Table 2) and spirometric indices in children from north India compared to other centres (Model 1) ii) potential impact of SEC on lung function from Indian-based studies (Model 2) iii) all remaining datasets with proportional reductions in FEV_1_ and FVC (Model 3).

After extensive analyses, preliminary GLI-adjustments were derived for the three models (S3 Table). Details of these preliminary models and data fit are presented in the S1 File, section 2.1 to facilitate future investigation and validation by any researchers who are able to collect high quality data from healthy South-Asian children.

## Discussion

In this study we collated spirometry data from 8,124 subjects from four Indian-based and five UK-based studies of South-Asian children. After adjustment for age, sex and height using the GLI equations for White Europeans, FEV_1_ was, on average, 15% lower in South-Asian children, with similar results for FVC emphasising the need for an ethnic adjustment when interpreting spirometry results from such children. By contrast, proportional reductions in FEV_1_ and FVC in the majority of datasets meant that the FEV_1_/FVC ratio, which is commonly used in the diagnosis of airway obstruction, remained independent of ethnic group. Despite the smaller lung volumes in relation to height observed across all datasets, there was marked variability between centres in the magnitude of both the mean offset from predicted values in White subjects (which ranged from 4–19%) and the between-subject variability (coefficient of variation). Although the existing ‘GLI-Other’ equation fitted data from North India reasonably well while the ‘Black’ ethnic adjustment provided a better approximation for most of the other South-Asian data than the ‘White equation’, the marked disparities between centres persisted, irrespective of which equation was applied.

These findings confirm previous observations by Aggarwal and colleagues who compared spirometry equations from different subsets of the Indian adult population and found that the three sets of equations (from North, South and West India) did not yield interchangeable results, suggesting that there may be considerable heterogeneity in predicted lung function in various Indian subgroups[31]. While several paediatric reference ranges for specific regions in India have been published, the applicability of these equations to other parts of India has yet to be tested.

### Strengths and limitations

Results from this study represent the largest collation of spirometry data from South-Asian children. Data were subjected to sophisticated statistical modelling which has been shown to be highly effective for developing all-age, multi-ethnic reference ranges[3]. However, the current data were collected for various purposes under a wide range of conditions, using different instrumentation and quality control with marked variability in the extent to which potential determinants of lung function had been documented. Thus, despite growing awareness of the potential effects of severe poverty [8,32–34], only two of the Indian-based studies reported such details. Similarly, lack of routine documentation regarding birth weight and gestational age in many regions within India, meant that children born prematurely or of low birthweight, both of which can have long term effects on lung function, could not be excluded from such studies. Since this was an opportunistic study based on retrospective data collection, we had no access to information on the extent of migration between rural and urban areas within India, nor details regarding ethnic sub-groups within the South-Asian population from several studies. The population included in this study was predominantly of Indian-origin and the relatively small sample size within other groups such as Pakistani and Bangladeshi precluded any definitive conclusions regarding the extent to which lung function may vary between such groups.

### How representative were the subjects recruited to this study?

The issue of which subjects to include in a reference population remains debateable[23,35]. We used an opportunistic approach by contacting authors who had recently submitted data from healthy South-Asian children to the GLI-initiative or who had published such data in the past 15 years, but did not attempt to collate data from South-Asian adults[5], this being beyond the remit of the current study.

All data collated were community based, with most studies specifically designed to recruit from a random sample of schools which catered to a wide range of socio-economic circumstances to ensure a representative sample. Although the Hyderabad data were collected before publication of modern standards for paediatric spirometry[14] which could have introduced some bias, as discussed below, this dataset was excluded when modelling the new GLI-adjustments, so will not have influenced our overall findings.

To assess how representative the data were, we also compared anthropometric data to the current Indian growth charts, derived from 18,666 children attending 10 affluent schools from five major geographical regions of India[29]. After adjusting for age and sex, anthropometric data from all but three centres (A, B and D) were comparable, with mean(SD) values ~0 (1), suggesting that the socio-demographics of most subjects included in this study were representative of well-nourished South-Asian children throughout India and the UK. As previously reported from a study of Punjabi children[36], South-Asian children from Northern India (Centre B) were taller and heavier than those from other centres. Whereas both anthropometry and lung function were lower in children from semi-urban/rural areas when compared with their urban counterparts in centre A, height and weight z-scores remained significantly lower in subjects from Centre D (Hyderabad) despite excluding all data from subjects from middle/low SEC, potential reasons for which could not be ascertained retrospectively.

#### Variability between and within centres

One of the most striking findings in this study was the variation in mean lung function between different centres. While FEV_1_ and FVC were on average 15% lower than that in White children of similar age and height, this difference ranged from 4–19% across the datasets. While mean differences of up to 0.5 z-scores (~5%) in lung function can occur by chance in small samples[37], this could not account for differences between some of the larger centres included in this study. Similarly, there were marked differences in within-centre, between-subject variability. While not necessarily a reason for exclusion per se, inclusion of datasets with markedly increased coefficient of variation when deriving reference equations may decrease their sensitivity to detect lung disease[38]. Review of the literature reveals a similar degree of heterogeneity within the South-Asian population[5,7,39], reasons for which need urgent elucidation if reliable reference ranges are to be developed.

#### Methodological issues

An important factor contributing to differences in both mean values between centres and scatter of results within-centres may relate to equipment used. One possible contributory explanation for differences between Centre G and other centres could be systematic overestimation by the spirometer despite daily calibration, since the relative differences in FEV_1_ and FVC between South-Asian and White children in this study were similar to other reports. While all centres strived to achieve international ATS/ERS standards, the quality of data will inevitably be influenced by the training, expertise and experience of the technical staff. Unfortunately, the lack of stored flow-volume curves with which to assess quality of spirometry data from six of nine centres(Table 1) meant that there were no objective criteria to separate valid from invalid measurements unless data were physiologically impossible. In contrast to some studies wherein strict QC was precluded by the type of spirometer used[5], Centres A (Bangalore) and I (SLIC study) both used a modern ultrasonic device which stores all data. This enabled immediate inspection of flow-volume data at time of collection as well as subsequent over-read, both essential features when assessing children[7,40]. Following quality control, this equipment also allows automatic upload of results into a research database, precluding transcription errors that may occur during manual entry.

In the current study, mean zFEV_1_/FVC from all but two centres approximated zero (Table 2). When studying healthy children, a relatively low FVC combined with a normal or high FEV_1_/FVC ratio, especially if combined with a wide scatter of results, may reflect poor subject cooperation, rather than lung restriction[41,42]. Additional discrepancies will have been introduced in several studies by failure to record age to at least 1 decimal year (Fig 1), the importance of which for accurate prediction of lung function was emphasised recently[3].

#### Impact of socio-economic circumstances

The marked secular changes in both lung function and growth that have occurred within developing nations such as India over the past 50 years[43] may contribute to increased variability of results in South-Asians, especially if exposed to extreme social deprivation[7], but are less likely to explain discrepancies in studies undertaken in the UK[9,26]. Despite minimal impact of SEC on lung function in children living in the UK[9,11,44], we recently reported a potential threshold effect of poverty, with lung function being significantly lower in children residing in rural India than in their urban counterparts[7], which is in keeping with previous evidence[8]. In an attempt to avoid either over-diagnosis of lung disease in otherwise healthy children with evidence of growth restriction or poorer living conditions, or under-diagnosis in well-nourished children, separate GLI-adjustment factors were derived for these groups.

**Regional differences?** The extent to which inclusion of various South-Asian subgroups contributed to both heterogeneity of lung function within studies and differences between centres is difficult to ascertain due to sample size and inconsistency in the way in which such details were recorded. Despite inevitable mixing, people from north India have different ancestry from those in the south[45,46] which impacts on both anthropometry and lung function, and which may account for the higher lung function for data collected in Delhi[47]. Consequently, a separate preliminary equation was derived for this dataset.

While it is customary to use stature as a proxy for lung volume, this approach does not adjust for frame size including leg length in relation to torso (i.e. sit/standing height ratio). We have not reported detailed anthropometry regarding sit/stand ratio or chest dimensions in this report, as so few studies have included such measures, and those that did found that they made a relatively small contribution to observed differences between White and South-Asian children[9,26]. Nevertheless, while absolute lung volumes adjusted for stature may differ between ethnic groups, there is strong evidence that among healthy individuals, such differences in FEV_1_ and FVC are proportional, as observed in over 60 datasets submitted to the GLI-2012[30], resulting in an FEV_1_/FVC ratio that is relatively independent of ethnicity and body size[3,23]. Datasets with non-proportional reductions in FEV_1_ and FVC based on the ‘White equation’ were excluded prior to deriving South-Asian GLI-adjustment factors since not only may an elevated FEV_1_/FVC in health reflect difficulties in achieving a full exhalation, but such findings are contrary to the majority of data previously published or submitted to the GLI.

### Clinical and practical implications

While this study has provided clear evidence of ethnic differences in spirometric lung function among South- Asian children, it must be emphasised that the proportionally smaller values of FEV_1_ and FVC observed do not reflect intrinsic differences in the functionality of the respiratory system, as reflected by the consistency of the FEV_1_/FVC ratio. While derivation of an ethnic GLI-adjustment would potentially be the most efficient and cost-effective way of accommodating those of South-Asian origin as has been done for those of Black-African origin, results from this study suggest that no single adjustment will currently fit all data collected from this group, since there appear to be complex factors contributing to the heterogeneity observed within the South-Asian population. While it may be possible to address some of these factors in future, for example by greater standardisation of equipment, methodology and quality control, whereas the contribution of others, such as extreme poverty, may diminish over time with improved living standards, there may be some intrinsic differences related to genetic ancestry, which need further investigation. Meanwhile, the clinical dilemma of which equations to choose when interpreting spirometry results from South-Asian children remains. Personal communication with the authors from the Indian based studies included in this initiative, revealed that within most clinical centres in India, technicians frequently rely on the unspecified and disparate reference values presented as default by the manufacturers, a practice not unknown even in the UK[1]. Under these circumstances, adoption of a common standard such as the GLI all-age equations which are already available in most commercial lung function equipment (http://www.ers-education.org/guidelines/global-lung-function-initiative.aspx) would be far preferable, even if this did entail a small offset. Results from the current study suggest that until an improved solution can be found, use of the GLI-Black equations for the majority of South-Asian children, or the GLI-other equation for those originating from North India, could provide an interim solution. For any clinical centre with access to a sizeable population (ideally at least 300[37]) of healthy children, use of such equations could be validated and if necessary the lower limit of normal adjusted to the local population, as discussed above (Table 7; see also S1 File, section 2.1.1, S6 Table).

Given that such an approach will need updating in future, we have included three alternative equations in the S1 File, in the hopes that this might stimulate further research using high quality equipment and standardised protocols to investigate the potential impact that SEC, geographical location or ethnic sub-groups may impact anthropometry and lung function within the South-Asian. When attempting to derive any new adjustment for South Asians, there needs to be a balance between limited generalisability if equations are based on a few homogenous datasets collected under ideal circumstances (which may not be achievable during routine clinical assessments) versus the lack of discrimination that will occur if such equations are derived from extremely heterogeneous data.

### Conclusions and Future Directions

Variations in both the mean level of lung function and the degree of within-subject variability meant that none of the existing GLI-ethnic adjustments provided an ideal fit for all the datasets examined in this study and also precluded derivation of a single new South-Asian paediatric GLI adjustment. Given the current wide variation in equations used to interpret lung function from South-Asian children, an alternative approach would be to express results from South-Asian children using either the ‘GLI-Black’ or ‘GLI -Other’ equations, depending on region of origin, both of which are widely available in current spirometers.

Further prospective data collection among South-Asians residing within the Indian sub-continent as well as those who have migrated, using standardised protocols over a wider age range, is required to explore the potential sources of variation observed, with regular updating if there is evidence of significant secular changes. When undertaking such studies, age should be recorded in decimal years and standardised anthropometry include assessments of sitting height. All lung function assessments need to be undertaken according to ATS/ERS guidelines[14], adapted for children where necessary[40] using equipment that allows prospective QC at time of data collection, storage of all data for subsequent independent over-read and automated export of results to avoid transcription errors. Furthermore, documentation of relevant past and current medical history, environmental exposures and SEC should be as standardised as much as possible to facilitate subsequent collation or sub-division of data, and clarification of potential sources of variability (S8 Table).

In conclusion, despite some progress being made, the challenges we faced in attempting to derive spirometry reference equations for South-Asian children are perhaps best summarised by the following quote from Earl C. Kelley, Professor of Secondary Education at Wayne University (1951)[48]:

"We have not succeeded in answering all our problems—indeed we sometimes feel we have not completely answered any of them. The answers we have found have only served to raise a whole set of new questions. In some ways we feel that we are as confused as ever, but we think we are confused on a higher level and about more important things. So this report does not purport to give final answers, or to claim that we now "know how to do it". We see more need for revision than ever. But we are doing better than we did. And this is a progress report, rendered with humility because of the unsolved problems we see now which we could not see before."

## Supporting Information

S1 Fig Amendment to Excel Sheet calculator for calculation of lung function z-scores based on preliminary GLI-adjustments (for Model 3b). (PDF)

S2 Fig Data fit of the GLI-adjustment for Centre B using the smoothing function, plotted against height. (PDF)

S3 Fig FVC z-scores based on Model 2 (GLI-adjustments for A_2-3_ & C) according to centre. (PDF)

S4 Fig Distribution of lung function z-scores calculated using GLI-adjustment based on Model 3a. (PDF)

S5 Fig FVC z-scores calculated using Model 3a (GLI-adjustments for A1, E, F, H & I) according to centre. (PDF)

S6 Fig FVC z-scores based on Model 3b (GLI-adjustments for A_1_, H & I) according to centre. (PDF)

S1 File Supporting information. (PDF)

S1 Table Recruitment and exclusion criteria according to respective studies. (PDF)

S2 Table Comparison of anthropometry between children residing in the UK and in India. (PDF)

S3 Table Preliminary GLI-adjustments according to the various models. (PDF)

S4 Table Lung function results based on Model 1 GLI-coefficients derived from Centre B (Delhi). (PDF)

S5 Table Lung function results based on Model 2 GLI-coefficients derived from Centres A_2-3_ & C. (PDF)

S6 Table Lung function results based on GLI-coefficients derived from Centres A_1_(urban), E, F, H & I (Model 3a). (PDF)

S7 Table Lung function results based on Model 3b GLI-coefficients derived from Centres A_1_, H and I (PDF)

S8 Table Data required for prospective data collection. (PDF)

## References

[1] Stanojevic S, Wade A, Stocks J (2010) Reference values for lung function: past, present and future. Eur Respir J 36: 12–19. 10.1183/09031936.00143209 20595163

[2] Subbarao P, Lebecque P, Corey M, Coates AL (2004) Comparison of spirometric reference values. Pediatr Pulmonol 37: 515–522. 15114552 10.1002/ppul.20015

[3] Quanjer PH, Stanojevic S, Cole TJ, Baur X, Hall GL, Culver BH, et al (2012) Multi-ethnic reference values for spirometry for the 3-95-yr age range: the global lung function 2012 equations. Eur Respir J 40: 1324–1343. 10.1183/09031936.00080312 22743675 PMC3786581

[4] Pellegrino R, Viegi G, Brusasco V, Crapo RO, Burgos F, Casaburi R, et al (2005) Interpretative strategies for lung function tests. Eur Respir J 26: 948–968. 16264058 10.1183/09031936.05.00035205

[5] Duong M, Islam S, Rangarajan S, Teo K, O'Byrne PM, Schunemann HJ, et al (2013) Global differences in lung function by region (PURE): an international, community-based prospective study. Lancet Respir Med 1: 599–609. 10.1016/S2213-2600(13)70164-4 24461663

[6] Burney PG, Hooper RL (2012) The use of ethnically specific norms for ventilatory function in African-American and white populations. Int J Epidemiol 41: 782–790. 10.1093/ije/dys011 22434864 PMC3396311

[7] Sonnappa S, Lum S, Kirkby J, Bonner R, Wade A, Subramanya V, et al (2015) Disparities in pulmonary function in healthy children across the Indian urban-rural continuum. Am J Respir Crit Care Med 191: 79–86. 10.1164/rccm.201406-1049OC 25412016 PMC4299630

[8] Raju PS, Prasad KVV, Ramana YV, Balakrishna N, Murthy KJ (2005) Influence of socioeconomic status on lung function and prediction equations in Indian children. Pediatr Pulmonol 39: 528–536. 15789442 10.1002/ppul.20206

[9] Lum S, Bountziouka V, Sonnappa S, Wade A, Cole TJ, Harding S, et al (2015) Lung function in children in relation to ethnicity, physique and socioeconomic factors. Eur Respir J 46: 1662–1671. 10.1183/13993003.00415-2015 26493801 PMC4668600

[10] Strippoli MP, Kuehni CE, Dogaru CM, Spycher BD, McNally T, Silverman M, et al (2013) Etiology of ethnic differences in childhood spirometry. Pediatrics 131: e1842–1849. 10.1542/peds.2012-3003 23713103

[11] Whitrow MJ, Harding S (2008) Ethnic differences in adolescent lung function: anthropometric, socioeconomic, and psychosocial factors. Am J Respir Crit Care Med 177: 1262–1267. 10.1164/rccm.200706-867OC 18323540 PMC2643205

[12] Menezes AM, Wehrmeister FC, Hartwig FP, Perez-Padilla R, Gigante DP, Barros FC, et al (2015) African ancestry, lung function and the effect of genetics. Eur Respir J 45: 1582–1589. 10.1183/09031936.00112114 25700383 PMC4450153

[13] Harik-Khan RI, Fleg JL, Muller DC, Wise RA (2001) The effect of anthropometric and socioeconomic factors on the racial difference in lung function. Am J Respir Crit Care Med 164: 1647–1654. 11719304 10.1164/ajrccm.164.9.2106075

[14] Miller MR, Hankinson J, Brusasco V, Burgos F, Casaburi R, Coates A, et al (2005) Standardisation of spirometry. Eur Respir J 26: 319–338. 16055882 10.1183/09031936.05.00034805

[15] Chhabra SK, Kumar R, Gupta U, Rahman M, Dash DJ (2014) Prediction equations for spirometry in adults from northern India. Indian J Chest Dis Allied Sci 56: 221–229. 25962195

[16] Memon MA, Sandila MP, Ahmed ST (2007) Spirometric reference values in healthy, non-smoking, urban Pakistani population. J Pak Med Assoc 57: 193–195. 17489528

[17] Chhabra SK, Vijayan VK, Rahman M, Mittal V, Singh PD (2012) Regression equations for spirometry in children aged 6 to 17 years in Delhi region. Indian J Chest Dis Allied Sci 54: 59–63. 22779126

[18] Doctor TH, Trivedi SS, Chudasama RK (2010) Pulmonary function test in healthy school children of 8 to 14 years age in south Gujarat region, India. Lung India 27: 145–148. 10.4103/0970-2113.68317 20931033 PMC2946716

[19] Budhiraja S, Singh D, Pooni PA, Dhooria GS (2010) Pulmonary functions in normal school children in the age group of 6–15 years in north India. Iran J Pediatr 20: 82–90. 23056687 PMC3445999

[20] Prasad R, Verma SK, Agrawal GG, Mathur N (2006) Prediction model for peak expiratory flow in North Indian population. Indian J Chest Dis Allied Sci 48: 103–106. 16696523

[21] Raju PS, Prasad KVV, Ramana YV, Murthy KJ (2004) Pulmonary function tests in Indian girls—prediction equations. Indian J Pediatr 71: 893–897. 15531830 10.1007/BF02830828

[22] Vijayan VK, Reetha AM, Kuppurao KV, Venkatesan P, Thilakavathy S (2000) Pulmonary function in normal south Indian children aged 7 to 19 years. Indian J Chest Dis Allied Sci 42: 147–156. 11089318

[23] Lum S, Bountziouka V, Sonnappa S, Cole TJ, Bonner R, Stocks J (2015) How "healthy" should children be when selecting reference samples for spirometry? Eur Respir J 45: 1576–1581. 10.1183/09031936.00223814 25700391 PMC4452263

[24] Kirkby J, Lum S, Stocks J, Bonner R, Sonnappa S (2014) Adaptation of the GLI-2012 spirometry reference equations for use in Indian children. European Respiratory Journal 44: 191.

[25] Raju PS, Prasad KVV, Ramana YV, Ahmed SK, Murthy KJ (2003) Study on lung function tests and prediction equations in Indian male children. Indian Pediatr 40: 705–711. 12951372

[26] Whittaker AL, Sutton AJ, Beardsmore CS (2005) Are ethnic differences in lung function explained by chest size? Arch Dis Child Fetal Neonatal Ed 90: F423–428. 15871993 10.1136/adc.2004.062497 PMC1721951

[27] Whincup PH, Nightingale CM, Owen CG, Rudnicka AR, Gibb I, McKay CM, et al (2010) Early emergence of ethnic differences in type 2 diabetes precursors in the UK: the Child Heart and Health Study in England (CHASE Study). PLoS Med 7: e1000263 10.1371/journal.pmed.1000263 20421924 PMC2857652

[28] Barone-Adesi F, Dent JE, Dajnak D, Beevers S, Anderson HR, Kelly FJ, et al (2015) Long-Term Exposure to Primary Traffic Pollutants and Lung Function in Children: Cross-Sectional Study and Meta-Analysis. PLoS One 10: e0142565 10.1371/journal.pone.0142565 26619227 PMC4664276

[29] Khadilkar VV, Khadilkar AV, Cole TJ, Sayyad MG (2009) Cross-sectional growth curves for height, weight and body mass index for affluent Indian children, 2007. Indian Pediatr 46: 477–489. 19556658

[30] Quanjer PH, Stanojevic S, Stocks J, Hall GL, Prasad KV, Cole TJ, et al (2010) Changes in the FEV(1)/FVC ratio during childhood and adolescence: an intercontinental study. Eur Respir J 36: 1391–1399. 10.1183/09031936.00164109 20351026

[31] Aggarwal AN, Gupta D, Jindal SK (2007) Comparison of Indian reference equations for spirometry interpretation. Respirology 12: 763–768. 17875069 10.1111/j.1440-1843.2007.01123.x

[32] Lawlor DA, Ebrahim S, Davey Smith G (2004) Association between self-reported childhood socioeconomic position and adult lung function: findings from the British Women's Heart and Health Study. Thorax 59: 199–203. 14985552 10.1136/thorax.2003.008482 PMC1746970

[33] Jackson B, Kubzansky LD, Cohen S, Weiss S, Wright RJ (2004) A matter of life and breath: childhood socioeconomic status is related to young adult pulmonary function in the CARDIA study. Int J Epidemiol 33: 271–278. 15082626 10.1093/ije/dyh003

[34] Stein CE, Kumaran K, Fall CH, Shaheen SO, Osmond C, Barker DJ (1997) Relation of fetal growth to adult lung function in south India. Thorax 52: 895–899. 9404378 10.1136/thx.52.10.895 PMC1758421

[35] Stanojevic S, Wade A, Lum S, Stocks J (2007) Reference equations for pulmonary function tests in preschool children: a review. Pediatr Pulmonol 42: 962–972. 17726704 10.1002/ppul.20691

[36] Singh SP, Sidhu LS, Malhotra P (1987) Growth performance of Punjabi children aged 6–12 years. Ann Hum Biol 14: 169–179. 3606028 10.1080/03014468700006892

[37] Quanjer PH, Stocks J, Cole TJ, Hall GL, Stanojevic S (2011) Influence of secular trends and sample size on reference equations for lung function tests. Eur Respir J 37: 658–664. 10.1183/09031936.00110010 20817707

[38] Stanojevic S, Wade A, Stocks J, Hankinson J, Coates AL, Pan H, et al (2008) Reference ranges for spirometry across all ages: a new approach. Am J Respir Crit Care Med 177: 253–260. 18006882 10.1164/rccm.200708-1248OC PMC2643211

[39] Khongsdier R (2001) Body mass index of adult males in 12 populations of northeast India. Ann Hum Biol 28: 374–383. 11459235 10.1080/03014460010004610

[40] Kirkby J, Welsh L, Lum S, Fawke J, Rowell V, Thomas S, et al (2008) The EPICure study: comparison of pediatric spirometry in community and laboratory settings. Pediatr Pulmonol 43: 1233–1241. 10.1002/ppul.20950 19009621

[41] Swanney MP, Beckert LE, Frampton CM, Wallace LA, Jensen RL, Crapo RO (2004) Validity of the American Thoracic Society and other spirometric algorithms using FVC and forced expiratory volume at 6 s for predicting a reduced total lung capacity. Chest 126: 1861–1866. 15596685 10.1378/chest.126.6.1861

[42] Aaron SD, Dales RE, Cardinal P (1999) How accurate is spirometry at predicting restrictive pulmonary impairment? Chest 115: 869–873. 10084506 10.1378/chest.115.3.869

[43] Quanjer PH, Kubota M, Kobayashi H, Omori H, Tatsumi K, Kanazawa M, et al (2015) Secular changes in relative leg length confound height-based spirometric reference values. Chest 147: 792–797. 10.1378/chest.14-1365 25254426

[44] Kuehni CE, Strippoli MP, Spycher BD, Silverman M, Beardsmore CS (2015) Lung function in the children of immigrant and UK-born south-Asian mothers. Eur Respir J 45: 1163–1166. 10.1183/09031936.00152114 25573409

[45] Ali M, Liu X, Pillai EN, Chen P, Khor CC, Ong RT, et al (2014) Characterizing the genetic differences between two distinct migrant groups from Indo-European and Dravidian speaking populations in India. BMC Genet 15: 86 10.1186/1471-2156-15-86 25053360 PMC4120727

[46] Majumder PP (1998) People of India: Biological diversity and affinities. Evolutionary Anthropology 6: 100–110.

[47] Chhabra SK (2009) Regional variations in vital capacity in adult males in India: comparison of regression equations from four regions and impact on interpretation of spirometric data. Indian J Chest Dis Allied Sci 51: 7–13. 19317357

[48] Kelley EC (1951) Workshop way of learning: Harper & Row.

