# Supplementary material for: Challenges in Collating Spirometry Reference Data for South-Asian Children: An Observational Study
Source: PLoS One. 2016 Apr 27;11(4):e0154336. doi: 10.1371/journal.pone.0154336 (PMC4847904; doi:10.1371/journal.pone.0154336)
Supplement: S1 Fig — (PDF) [file pone.0154336.s001.pdf]

S1 Fig. Amendment to Excel Sheet calculator for calculation of lung function z-scores based on preliminary GLI-adjustments (for Model 3b)

	A	B	C	D	E	F	G	H	I	J	K	L	M	N	O	P
1		FEV1						FVC						FEV1/FVC		
2		Male			Female			Male			Female			Male		
3	Age	L	mu.s	sigma.s	L	mu.s	sigma.s	L	mu.s	sigma.s	L	mu.s	sigma.s	L	mu.s	sigma.s
4	log link	0.00000	1.00000	1.00000	0.00000	1.00000	1.00000	0.00000	1.00000	1.00000	0.00000	1.00000	1.00000	0.00000	1.00000	1.00000
5	log ht	0.00000	1.00000	0.00000	0.00000	1.00000	0.00000	0.00000	1.00000	0.00000	0.00000	1.00000	0.00000	0.00000	1.00000	0.00000
6	coef int	0.88660	-10.34200	-2.32680	1.15400	-9.69870	-2.37650	0.94810	-11.22810	-2.29630	0.82360	-10.40300	-2.35490	4.71010	0.74030	-2.95950
7	Height cn	0.00000	2.21960	0.00000	0.00000	2.12110	0.00000	0.00000	2.41350	0.00000	0.00000	2.26330	0.00000	0.00000	-0.15950	0.00000
8	coef age	0.08500	0.05740	0.07980	0.00000	-0.02700	0.09720	0.00000	0.08650	0.07180	0.00000	0.02340	0.10170	-0.67740	-0.03660	0.11560
9	power age	0.00000	0.00000	0.00000	0.00000	0.00000	0.00000	0.00000	0.00000	0.00000	0.00000	0.00000	0.00000	0.00000	0.00000	0.00000
10	Afr. Am.	0.00000	-0.12940	0.10560	0.00000	-0.12940	0.10560	0.00000	-0.12240	0.08020	0.00000	-0.12240	0.08020	0.00000	-0.01350	-0.03440
11	NE Asia	0.00000	-0.03510	-0.39730	0.00000	-0.01490	-0.01090	0.00000	-0.04050	-0.46000	0.00000	-0.02620	-0.18090	0.00000	0.00550	-0.22270
12	SE Asia	0.00000	-0.08810	0.03270	0.00000	-0.12080	0.07330	0.00000	-0.11770	0.03250	0.00000	-0.15160	0.04590	0.00000	0.02830	-0.14140
13	O/M	0.00000	-0.07080	0.01140	0.00000	-0.07080	0.01140	0.00000	-0.08250	-0.05030	0.00000	-0.08330	-0.05030	0.00000	0.01060	-0.08600
14	3	0.00000	-0.11332	0.21434	0.00000	-0.23111	0.33515	0.00000	-0.09378	0.29861	0.00000	-0.19405	0.36935	1.38762	-0.02207	-0.08316
15	3.25	0.00000	-0.10726	0.20434	0.00000	-0.21700	0.30976	0.00000	-0.08881	0.27845	0.00000	-0.18241	0.34310	1.26209	-0.01914	-0.06165
