# Supplementary material for: Challenges in Collating Spirometry Reference Data for South-Asian Children: An Observational Study
Source: PLoS One. 2016 Apr 27;11(4):e0154336. doi: 10.1371/journal.pone.0154336 (PMC4847904; doi:10.1371/journal.pone.0154336)
Supplement: S3 Fig — (PDF) [file pone.0154336.s003.pdf]

S3 Fig. FVC z-scores based on Model 2 (GLI-adjustments for $A_{2\text{-}3}$ & C) according to centre

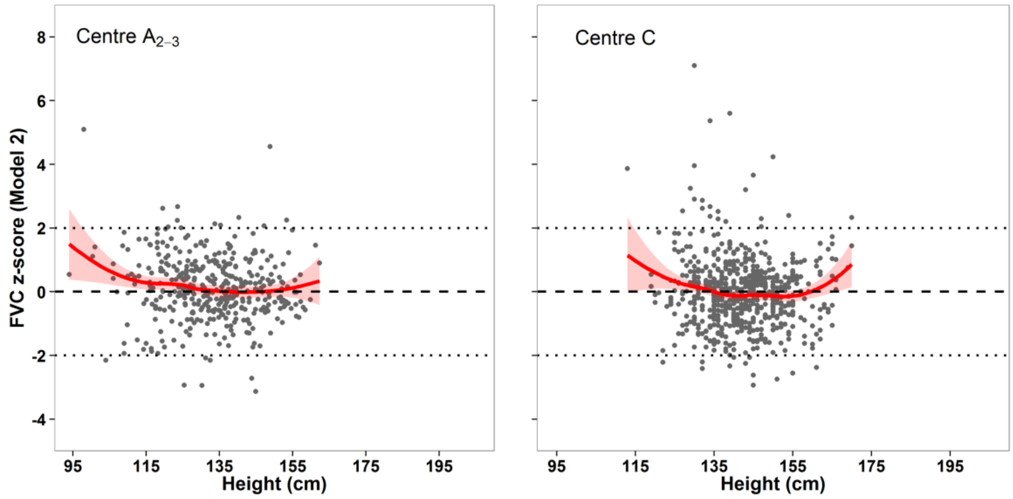

Legend: Individual data are shown for each centre. The dashed line denotes the predicted mean (0 z-score) and the dotted lines denote the upper and lower limit of the normal range which should encompass 95% of healthy subjects (±1.96 z-scores). The data fit according to the preliminary GLI-coefficient for children from each centre using the smoothing function is denoted by the red line, the 95% Confidence limits for which are represented by the pink shaded area. The wider 95% confidence limits at either end of the height distribution reflect the low sample size at these heights.
