# Supplementary material for: Challenges in Collating Spirometry Reference Data for South-Asian Children: An Observational Study
Source: PLoS One. 2016 Apr 27;11(4):e0154336. doi: 10.1371/journal.pone.0154336 (PMC4847904; doi:10.1371/journal.pone.0154336)
Supplement: S4 Fig — (PDF) [file pone.0154336.s004.pdf]

S4 Fig. Distribution of lung function z-scores calculated using GLI-adjustment based on Model 3a

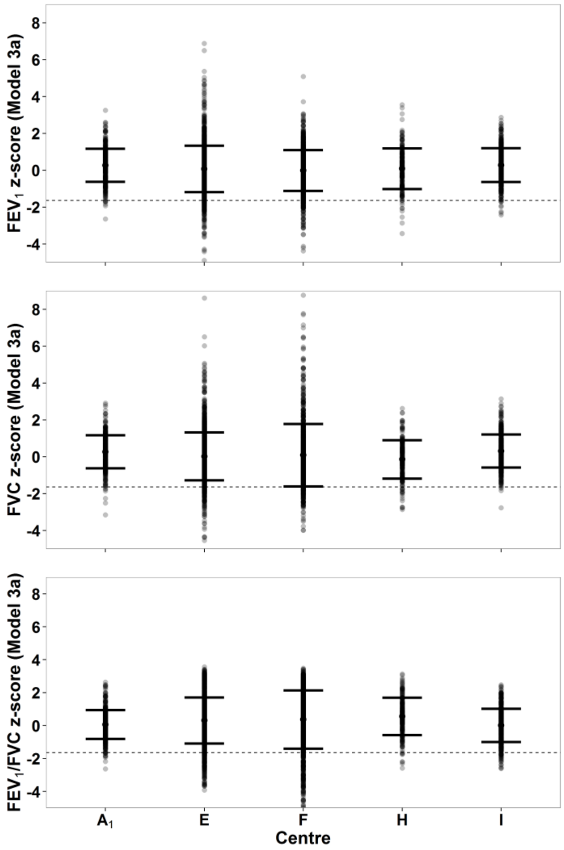

Legend: Data are presented as individual data points (black dots) with error bars to represent the mean (SD) for each centre. The dashed line denotes the lower limit of normal (i.e. LLN of -1.645 z-scores). Due to the marked spread of FVC z-score values from Centre G, the y-axis scale has been truncated and extreme values >8z-scores (n=4) have been omitted from the graph.
