# Supplementary material for: Challenges in Collating Spirometry Reference Data for South-Asian Children: An Observational Study
Source: PLoS One. 2016 Apr 27;11(4):e0154336. doi: 10.1371/journal.pone.0154336 (PMC4847904; doi:10.1371/journal.pone.0154336)
Supplement: S1 File — (PDF) [file pone.0154336.s007.pdf]

Challenges in collating spirometry reference data for South-Asian children: an observational study

Sooky Lum, Vassiliki Bountziouka, Philip Quanjer, Samatha Sonnappa, Angela Wade, Caroline Beardsmore, Sunil.K. Chhabra, Rajesh K. Chudasama, Derek G. Cook, Seeromanie Harding, Claudia E. Kuehni, K.V.V. Prasad, Peter H. Whincup, Simon Lee and Janet Stocks

Supporting information

1 Methods: additional information

1.1 South-Asian reference population: exclusion criteria

Recruitment and exclusion criteria according to centre are summarised in S1 Table. Participating centres were requested to only submit data from healthy South-Asian paediatric subjects. Data were excluded if:

- Gestational age <37 weeks
- Current or chronic respiratory disease
- Congenital abnormalities likely to impact on lung development

S1 Table. Recruitment and exclusion criteria according to respective studies

	**Recruitment criteria**	**Exclusion criteria for deriving reference population for this study**
Bangalore[1]	School children 5 to 12 years of age	Children with overt signs of illness on test day; those with current or chronic respiratory disease or significant congenital abnormalities likely to influence lung function
Delhi[2]	School children of North Indian origin, determined by mother tongue & parentage, aged 6 to 17y, screened by a health questionnaire and physical examination. Only “normal” children were assessed.	
Gujarat[3]	Studying in class V to VIII aged 8 to 14y during November 2007 to April 2008	Children with history of (h/o) febrile illness in the last 2 weeks, upper respiratory tract infections like symptoms in the past 2 weeks, acute or chronic respiratory disease, any major systemic disease like cardiac or renal problems, clinical significant anaemia, h/o drug intake which can affect lung function; any allergy; children with bone deformity of chest or spine and any muscular weakness, family h/o atopy, asthma or other chronic lung diseases.
Hyderabad[4,5]	Healthy children aged between 5 and 15y	Children with any respiratory disease or had recent history of respiratory infections.
CHASE[6]*	Primary school children aged 9 to 10y	Gestational age <37 w; Children with current or chronic respiratory disease or significant congenital abnormalities likely to influence lung function.
DASH[7]*	Children from Year 7 and 8 (11-13 years old)	Gestational age <37 w; current or chronic respiratory disease or significant congenital abnormalities likely to influence lung function;
Leicester city[8]*	Children aged 6-11 years from nine city primary schools	Children with a BMI >30kg/m^2, h/o cardio-pulmonary disease, chest wall deformity, or preterm delivery. Although Asthma was not an exclusion criterion unless the child required daily medication, children with a diagnosis of asthma were not included in the collated dataset.
Leicester Respiratory Cohort[9]*		Gestational age <37 w; Children with current or chronic respiratory disease or significant congenital abnormalities likely to influence lung function.
SLIC[10]*	School children between 5 and 12 years of age	Gestational age <37 w; Children with current or chronic respiratory disease or significant congenital abnormalities likely to influence lung function.

*Studies where recruitment criteria were broader due to their specific study aims but authors were requested to only submit data from healthy children (see exclusion criteria).

For the development of reference ranges, the following records were also excluded:

- missing data (e.g. height, FEV_1 or FVC)
- Implausible data (e.g. FEV_1/FVC >1; FEV_1 or FVC ≤0.3 L)

1.2 Data analyses and statistical methods

See main manuscript for full details.

GLI spirometry reference equations were available for the following ethnic and geographic groups:

- White Europeans (Caucasians, i.e. original peoples of Europe, Middle East or North Africa)
- Black-African origin (derived from data from African Americans [Afr.Am])
- South-East Asians (e.g. Thailand, Taiwan, China south of the Huaihe river and Qinling mountains)
- North-East Asians (e.g. Korea, China north of the Huaihe river and Qinling mountains)
- Other (consisting of groups other than the 4 main groups (above) and those of mixed ethnic origin)

GLI-spirometry reference equations for interpreting data from children originating from the Indian subcontinent (South-Asian) are currently not available.

The GLI-2012 data conversion software was used to derive GLI-adjustments for South-Asians[11] (http://www.ers-education.org/guidelines/global-lung-function-initiative/tools.aspx).

1.2.1 Application of preliminary GLI-adjustments for South Asians

The GLI-reference equations were derived using the LMS method, imbedded in GAMLSS which allows modelling the expected mean (M: [**M**u] predicted value), coefficient of variation (S: [**S**igma] scatter, which models the spread of values around the median and adjusts for any non-uniform dispersion) and an index of skewness (L: [**L**ambda] location)[12].

Provided the z-scores based on the GLI-White equations did not show any trend with age or height, signifying that the GLI model fit the data so that only proportional adjustments were required to fit a new group, adjustments for 'M' and 'S' were made using software provided by the GLI team. M was adjusted for a new group by calculating the sum of ln(y/M) in boys and girls, where y = measured and M the GLI predicted value for Whites, and dividing by the number of observations. The group specific adjustment factor for S was derived by taking the mean S (for boys and girls) of the ethnic subgroup (of the four included in the GLI published equations) that was closest to that seen in the new subgroup (i.e. GLI-Black).

The new GLI-adjustments for South-Asians (Models) were then used to convert data from each centre to z-scores using the GLI-2012 Excel Sheet calculator to ascertain how appropriate these were for each dataset with respect to mean values and distribution of data.

For researchers who wish to use the preliminary GLI-adjustments derived for South-Asian children, instructions are as follows:

Please download the following files from http://www.ers-education.org/guidelines/global-lung-function-initiative/tools.aspx

- Excel sheet calculator

- GLI-2012 Excel Sheet Calculator
- GLI-2012 Excel Sheet Calculator - Help file

Detailed instructions on how to apply the new GLI-coefficients are given in the Help file.

A brief summary as follows:

- Copy and paste the new coefficients "M" and "S" (from Models) into the relevant "mu.s" and "sigma.s" section of the "Afr.Am" group on Sheet 1 of the Excel Sheet calculator (Figure S1).
- Input the data onto sheet 2 of the excel sheet calculator and run the macro.
    - Please note: when inputting your data, since the new coefficients have been entered in the row for the "Afr.Am." ethnic group, then you will need to code the "Ethnic" variable as "Afr.Am." in sheet 2.

S1 Fig. Amendment to Excel Sheet calculator for calculation of lung function z-scores based on preliminary GLI-adjustments (for Model 3b)

	A	B	C	D	E	F	G	H	I	J	K	L	M	N	O	P
1		FEV1						FVC						FEV1/FVC		
2			Male			Female			Male			Female			Male	
3	Age	L	mu.s	sigma.s	L	mu.s	sigma.s	L	mu.s	sigma.s	L	mu.s	sigma.s	L	mu.s	sigma.s
4	log link	0.00000	1.00000	1.00000	0.00000	1.00000	1.00000	0.00000	1.00000	1.00000	0.00000	1.00000	1.00000	0.00000	1.00000	1.00000
5	log ht	0.00000	1.00000	0.00000	0.00000	1.00000	0.00000	0.00000	1.00000	0.00000	0.00000	1.00000	0.00000	0.00000	1.00000	0.00000
6	coef int	0.88660	-10.34200	-2.32680	1.15400	-9.69870	-2.37650	0.94810	-11.22810	-2.29630	0.82360	-10.40300	-2.35490	4.71010	0.74030	-2.95950
7	Height cn	0.00000	2.21960	0.00000	0.00000	2.12110	0.00000	0.00000	2.41350	0.00000	0.00000	2.26330	0.00000	0.00000	-0.15950	0.00000
8	coef age	0.08500	0.05740	0.07980	0.00000	-0.02700	0.09720	0.00000	0.08650	0.07180	0.00000	0.02340	0.10170	-0.67740	-0.03660	0.11560
9	power age	0.00000	0.00000	0.00000	0.00000	0.00000	0.00000	0.00000	0.00000	0.00000	0.00000	0.00000	0.00000	0.00000	0.00000	0.00000
10	Afr. Am.	0.00000	-0.12940	0.10560	0.00000	-0.12940	0.10560	0.00000	-0.12240	0.08020	0.00000	-0.12240	0.08020	0.00000	-0.01350	-0.03440
11	NE Asia	0.00000	-0.03510	-0.39730	0.00000	-0.01490	-0.01090	0.00000	-0.04050	-0.46000	0.00000	-0.02620	-0.18090	0.00000	0.00550	-0.22270
12	SE Asia	0.00000	-0.08810	0.03270	0.00000	-0.12080	0.07330	0.00000	-0.11770	0.03250	0.00000	-0.15160	0.04590	0.00000	0.02830	-0.14140
13	O/M	0.00000	-0.07080	0.01140	0.00000	-0.07080	0.01140	0.00000	-0.08250	-0.05030	0.00000	-0.08330	-0.05030	0.00000	0.01060	-0.08600
14	3	0.00000	-0.11332	0.21434	0.00000	-0.23111	0.33515	0.00000	-0.09378	0.29861	0.00000	-0.19405	0.36935	1.38762	-0.02207	-0.08316
15	3.25	0.00000	-0.10726	0.20434	0.00000	-0.21700	0.30976	0.00000	-0.08881	0.27845	0.00000	-0.18241	0.34310	1.26209	-0.01914	-0.06165

1.2.2 To ascertain appropriateness of GLI-adjustments to specific datasets

If the new ethnic adjustments for South-Asian children are appropriate, the group mean(SD) z-scores for data from each centre should approximate 0(1) across the entire age and height range studied, with no trend in the residuals[13]. In addition, the appropriateness of any given reference equation to specific datasets was ascertained by checking the percentage of healthy subjects within each centre with results that fell at or below the 5th centile (i.e. 5% lower limit of normal (LLN) ≤1.645 z-scores).

Lung function z-scores from all centres were also plotted against height and age separately and a smoothed curved line was fitted to the data using the loess (locally weighted scatterplot smoothing) procedure to ascertain the fit of the South-Asian GLI-adjustment to the data[14].

2 Results

Group characteristics and anthropometry of the collated data according to centre are presented in Table 2, main manuscript. When anthropometry was compared between South-Asian children residing in the UK to those in India, children in the UK were significantly taller and heavier compared to their Indian counterparts (S2 Table).

S2 Table. Comparison of anthropometry between children residing in the UK and in India

	UK	India	Mean(95%CI) difference (UK-India)
N (% boys)	3484 (52.1%)	4640 (59.6%)	-7% (-10%; -5%)***
Age (y)	10.6 (1.7)	10.3 (2.9)	0.3 (0.2; 0.4)***
zHeight	0.20 (1.02)	-0.36 (1.14)	0.56 (0.51; 0.61)***
zWeight	0.24 (1.03)	-0.60 (1.06)	0.84 (0.80; 0.89)***

Data presented as Mean (SD) unless otherwise specified. *** p< 0.0001; Height and weight were expressed as z-scores according to the Indian reference standard, which was based on well-nourished children.[15]

2.1 Derivation of new GLI-adjustments for South-Asian children

Preliminary GLI-adjustments (S3 Table) were derived based on the following rationale:

Model 1 (Centre B): which took into account the significantly higher anthropometric (Table 2, main manuscript) and spirometric indices in children recruited from Delhi (north India) compared to other centres

Model 2 (Centres $A_{2\text{-}3}$ & C): despite lacking details regarding SEC for the data from Gujarat (C), results were remarkably similar to those collected from children residing in semi-urban/rural Bangalore ($A_{2\text{-}3}$; Tables 2, 3). These datasets were therefore combined.

Model 3 (Centres A_1, E, F, H and I): i.e. all remaining datasets with similar mean offsets for FEV_1 and proportional reductions in FEV_1 and FVC.

S3 Table. Preliminary GLI-adjustments according to the various models

	FEV_1		FVC		FEV_1/FVC	
Centres	M	S	M	S	M	S
Model 1 (B)	-0.0853	0.1056	-0.0690	0.0802	-0.0210	-0.0344
Model 2 ($A_{2\text{-}3}$, C)	-0.2108	0.1056	-0.2089	0.0802	0.0032	-0.0344
Model 3a (A_1,E,F,H,I)	-0.1518	0.1056	-0.1432	0.0802	-0.0147	-0.0344
Model 3b (A_1,H,I)	-0.1294	0.1056	-0.1224	0.0802	-0.0135	-0.0344

Abbreviations: M=**M**u (median) or predicted value; S=**S**igma (coefficient of variation), which models the spread of values around the median and adjusts for any non-uniform dispersion.
Centres: A_1=Bangalore, urban; $A_{2\text{-}3}$=Bangalore, semi-urban & rural; B=Delhi; C=Gujarat; E=CHASE; F=DASH; H=Leicester Respiratory Cohort; I=SLIC; Model 3b: final/definitive model. The values of M indicate that when compared with the GLI reference for White subjects (calculated as 100*(1-exp (M)), FEV_1 and FVC were on average ~7% lower for Model 1; ~19% lower for Model 2 and ~12% lower for Model 3b with a relatively constant FEV_1/FVC across the models (Model 1: 2%; Model 2: 0.3%; Model 3a: 1.5%; Model 3b: 1.3%).
See above section 1.2.1 for details on how to apply these preliminary GLI-adjustments.

Model 1 (B: Delhi): After application of the GLI-adjustment derived from Model 1, the group mean (SD) for all spirometry outcomes from Centre B approximated 0(1) with 4.2% of children having an FEV_1/FVC below the LLN (≤-1.645 z-score) (S4 Table) and a good fit of the lung function z-scores (i.e. no trend observed in residuals) when plotted against either height (S2 Fig) or age (data not shown).

S2 Fig. Data fit of the GLI-adjustment for Centre B using the smoothing function, plotted against height

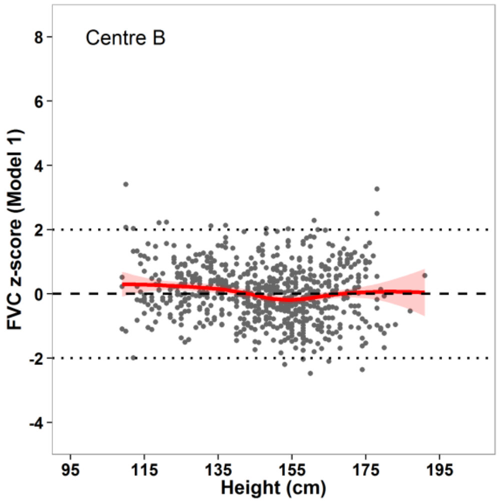

Legend: Individual data are shown for Centre B. The dashed line denotes the predicted mean (0 z-score) and the dotted lines denote the upper and lower limit of the normal range which should encompass 95% of healthy subjects (±1.96 z-scores). The data fit according to the preliminary GLI-coefficient for children from Delhi (B) using the smoothing function is denoted by the red line, the 95% Confidence limits (95% CI) for which are represented by the pink shaded area. The wider 95% confidence limits at either end of the height distribution reflect the small number of subjects at these heights.

S4 Table. Lung function results based on Model 1 GLI-coefficients derived from Centre B (Delhi)

Centre	n	$zFEV_1$	zFVC	$zFEV_1/FVC$	% ≤LLN $zFEV_1$	% ≤LLN zFVC	% ≤LLN $zFEV_1/FVC$
B	670	0.02 (0.86)	0.02 (0.89)	0.09 (1.06)	2.1%	2.2%	4.2%

Data presented as Mean (SD) unless otherwise specified. Abbreviations: LLN: Lower limit of normal (equates to ≤ -1.645 z-scores)

Model 2 (A_{2-3}&C): Similarly after deriving an GLI-adjustment from collated data from Bangalore (semi-urban/rural) & Gujarat and using this to derive lung function z-scores for these centres, a good fit was observed (S5 Table; S3 Fig).

S5 Table. Lung function results based on Model 2 GLI-coefficients derived from Centres A_{2-3} & C

Centre	n	$zFEV_1$	zFVC	$zFEV_1/FVC$	% ≤LLN $zFEV_1$	% ≤LLN zFVC	% ≤LLN $zFEV_1/FVC$
A_{2-3}	399	0.03(0.94)	0.11(1.01)	-0.18(0.93)	3.3%	4.3%	3.3%
C	648	0.07(1.02)	-0.03(1.09)	0.10(0.89)	3.4%	4.6%	3.1%
Total	1047	0.05(0.99)	0.02(1.06)	-0.01(0.91)	3.3%	4.5%	3.2%

Data presented as Mean (SD) unless otherwise specified. Abbreviations: LLN: Lower limit of normal (i.e. 5th centile which equates to ≤ -1.645 z-scores). Centre A_{2-3}: Bangalore (semi-urban & rural); Centre C: Gujarat

S3 Fig. FVC z-scores based on Model 2 (GLI-adjustments for $A_{2\text{-}3}$ & C) according to centre

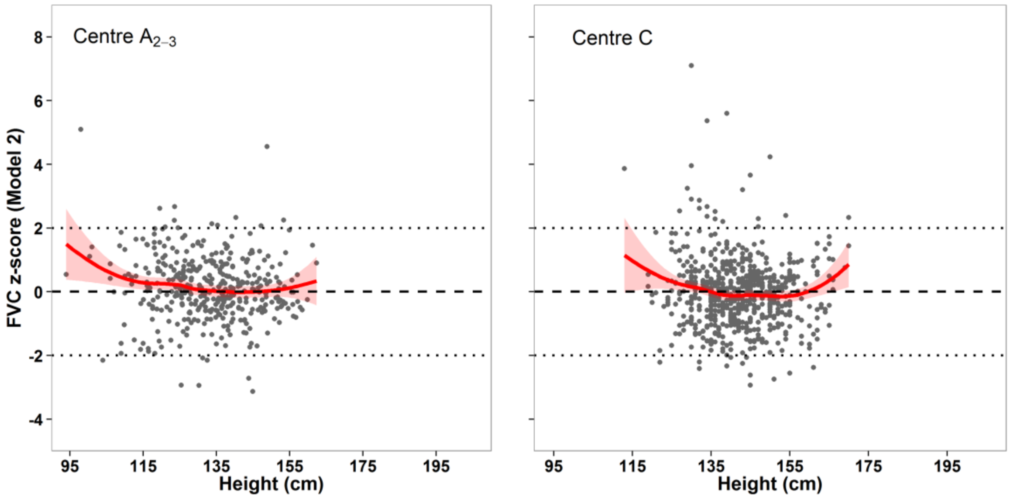

Legend: Individual data are shown for each centre. The dashed line denotes the predicted mean (0 z-score) and the dotted lines denote the upper and lower limit of the normal range which should encompass 95% of healthy subjects (±1.96 z-scores). The data fit according to the preliminary GLI-coefficient for children from each centre using the smoothing function is denoted by the red line, the 95% Confidence limits for which are represented by the pink shaded area. The wider 95% confidence limits at either end of the height distribution reflect the low sample size at these heights.

Model 3: By contrast, although group mean z-scores for all centres approximated zero, when GLI-adjustments derived from the remaining centres (A_1, E,F, H and I: Model 3a) were applied to the respective datasets, the spread of results was very high for Centres E and F, especially for zFVC and $zFEV_1$/FVC (S4 & S5 Figs). Furthermore, in contrast to the expected 5%, the proportion of children with an apparently "abnormal" result (i.e. ≤LLN) ranged from 1-13% according to outcome and centre (S6 Table).

S6 Table. Lung function results based on GLI-coefficients derived from Centres A_1(urban), E, F, H & I (Model 3a)

Centres	N	$zFEV_1$	zFVC	$zFEV_1$/FVC	%≤LLN $zFEV_1$	%≤LLN zFVC	% ≤LLN $zFEV_1$/FVC	Adj LLN‡ $zFEV_1$	Adj LLN‡ zFVC	Adj LLN‡ $zFEV_1$/FVC
A_1	383	0.27(0.90)	0.27(0.90)	0.06(0.88)	1.8%	1.6%	2.9%	-1.20	-1.24	-1.39
E	1547	0.07(1.26)	0.02(1.30)	0.31(1.40)	6.2%	6.9%	8.3%	-1.79	-1.84	-2.06
F	1064	-0.01(1.11)	0.08(1.69)	0.37(1.77)	7.0%	8.6%	13.4%	-1.83	-2.01	-3.20
H	210	0.09(1.11)	-0.14(1.04)	0.56(1.14)	4.3%	7.6%	3.8%	-1.63	-1.91	-1.31
I	486	0.28(0.92)	0.31(0.90)	0.01(1.01)	1.6%	0.8%	5.6%	-1.21	-1.10	-1.72
Total	3690	0.10(1.14)	0.09(1.34)	0.28(1.42)	5.3%	6.1%	8.6%	-1.67	-1.77	-2.20

Data presented as Mean (SD) unless otherwise specified. Abbreviations: LLN: Lower limit of normal (equates to ≤-1.645 z-scores); Adj LLN‡: LLN adjusted for the actual 5th centile according to each centre. Centres: A_1= Bangalore (urban); E=CHASE; F=DASH; H=Leicester Respiratory Cohort; I= SLIC

S4 Fig. Distribution of lung function z-scores calculated using GLI-adjustment based on Model 3a

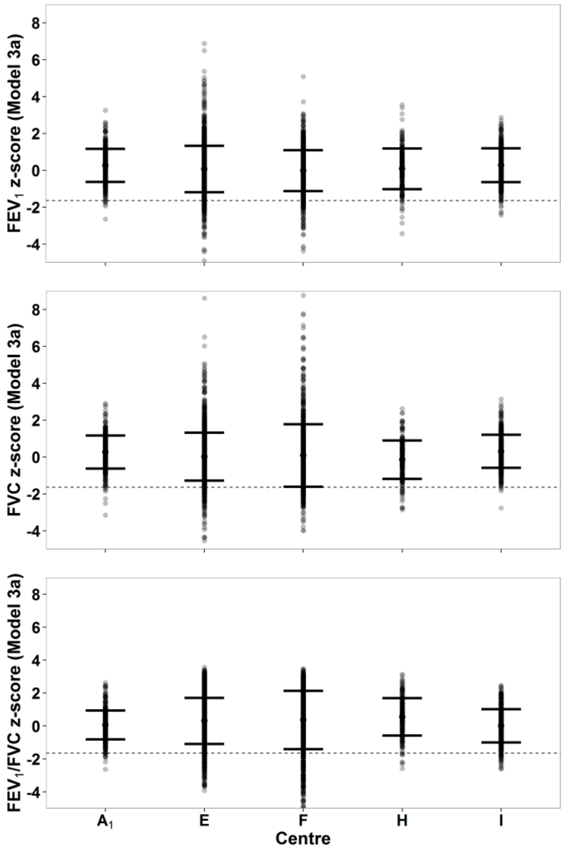

Legend: Data are presented as individual data points (black dots) with error bars to represent the mean (SD) for each centre. The dashed line denotes the lower limit of normal (i.e. LLN of -1.645 z-scores). Due to the marked spread of FVC z-score values from Centre G, the y-axis scale has been truncated and extreme values >8z-scores (n=4) have been omitted from the graph.

S5 Fig. FVC z-scores calculated using Model 3a (GLI-adjustments for A1, E, F, H & I) according to centre

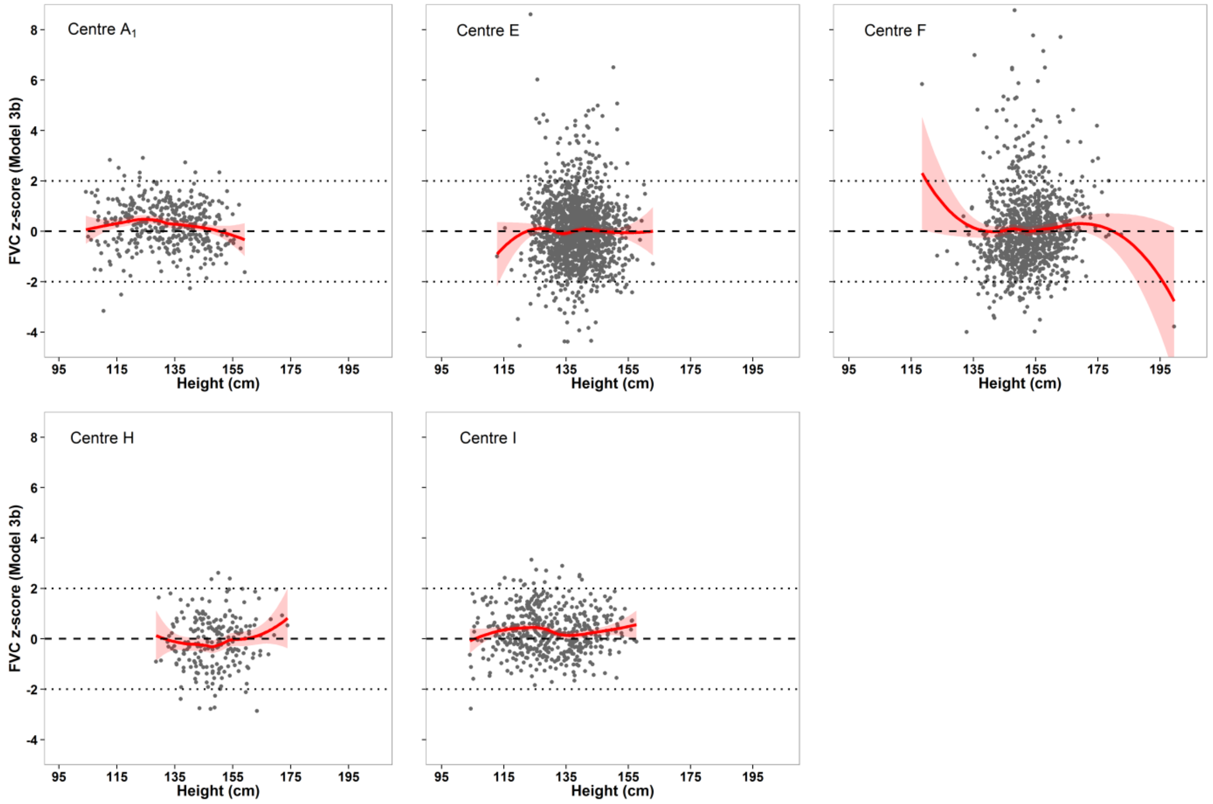

Individual data are shown for each centre. The dashed line denotes the predicted mean (0 z-score) and the dotted lines denote the upper and lower limit of the normal range which should encompass 95% of healthy subjects (±1.96 z-scores). The data fit according to the preliminary GLI-coefficient for children from each centre using the smoothing function is denoted by the red line, the 95% Confidence limits for which are represented by the pink shaded area. The wider 95% confidence limits at either end of the height distribution reflect the small number of subjects at these heights.

Since this could result in significant under- or over-diagnosis respectively of lung disease, data from E and F were excluded before recalculating a GLI-adjustment for the remaining centres (Model 3b:A_1, H & I). Although Model 3b provided a good fit for data from centres A_1 and I with between 1.5% to 5.3% of data falling ≤LLN and no trend in residuals, it was less appropriate for the smaller dataset from Centre H (S7 Table; S6 Fig).

S7 Table. Lung function results based on Model 3b GLI-coefficients derived from Centres A_1, H and I

Centre	n	$zFEV_1$	zFVC	$zFEV_1$/FVC	%≤ LLN $zFEV_1$	%≤LLN zFVC	% ≤ LLN $zFEV_1$/FVC
A_1	383	0.09(0.88)	0.11(0.88)	0.05(0.87)	2. 3%	2.6%	2.9%
H	210	-0.09(1.08)	-0.30(1.02)	0.54(1.13)	5.2%	9.0%	3.8%
I	486	0.10(0.89)	0.15(0.88)	-0.01(1.00)	2.5%	1.2%	5.6%
Total	1079	0.06(0.93)	0.05(0.92)	0.12(1.01)	3.0%	3.2%	4.3%

Data presented as Mean (SD) unless otherwise specified. Abbreviations: LLN: Lower limit of normal (equates to ≤ -1.645 z-scores). Centre A_1: Bangalore (urban); Centre H: Leicester Respiratory Cohort; Centre I: SLIC

S6 Fig. FVC z-scores based on Model 3b (GLI-adjustments for A_1, H & I) according to centre

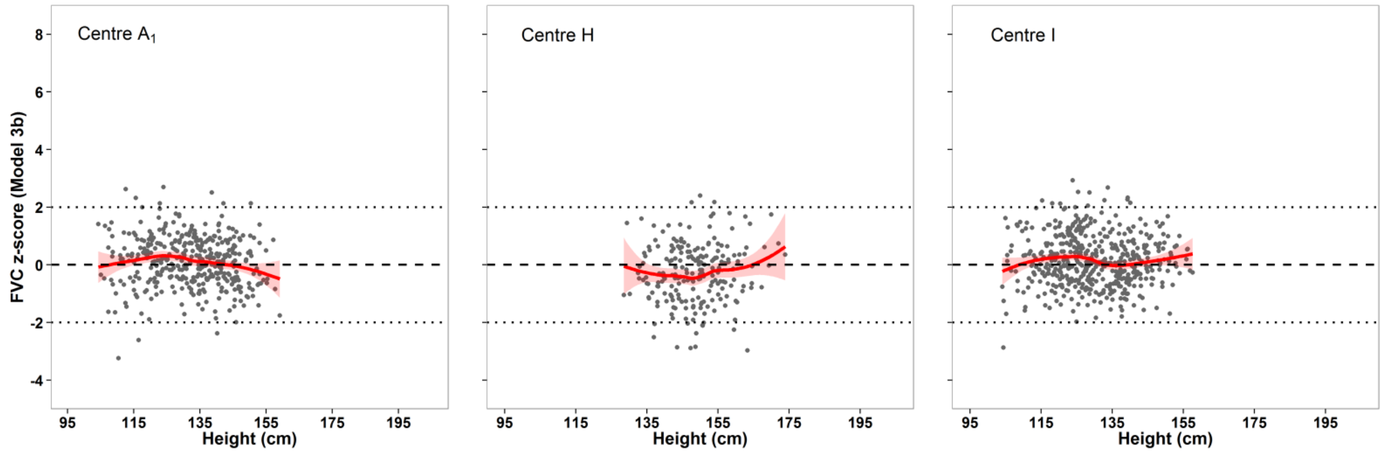

Legend: Individual data are shown for each centre. The dashed line denotes the predicted mean (0 z-score) and the dotted lines denote the upper and lower limit of the normal range which should encompass 95% of healthy subjects (±1.96 z-scores). The data fit according to the preliminary GLI-coefficient for children from each centre using the smoothing function is denoted by the red line, the 95% Confidence limits for which are represented by the pink shaded area. The wider 95% confidence limits at either end of the height distribution reflect the low sample size at these heights.

2.1.1 Use of adjusted lower limit of normal (Adj LLN)

When GLI-adjustments derived from the remaining centres (A_1, E, F, H and I: Model 3a) were applied to the respective datasets, group mean z-scores for all the centres generally approximated zero. However, the spread of results (SDs) varied markedly, being relatively low in centres A_1 and I and unusually high for Centre F with respect to both zFVC and zFEV$_1$/FVC. A similar pattern, though less marked was noted for Centre E (S2 Table, S3 Fig and Fig 3, main manuscript). In addition, the proportion of subjects in whom an “abnormal” zFEV$_1$/FVC was observed (≤ lower limit of normal [LLN] i.e. -1.645 z-scores) ranged from only ~1% in 3 centres to >10% in another (S2 Table). Thus for many of the centres, the LLN based on Model 3a (which for a healthy population should identify ~5% of results below the 5th centile) was inappropriate and could result in significant under or over-diagnosis respectively of lung disease. For this model (3a) to be applicable for all of the centres, it would be necessary to adjust the LLN for each outcome to fit the actual 5th centile observed for each centre (S2 Table). If using this approach, FEV$_1$ would be considered ‘abnormal’ if it was less than -1.21 (95%CI: -1.33; -1.11) z-score for a child studied in Centre I, whereas for one studied in centre F, the appropriate cut-off would be -1.83 (-1.99; -1.67) z-score. Similarly for FVC and FEV$_1$/FVC, the appropriate cut-offs would be -1.10 (-1.26; -0.95) and -1.72(-1.77; -1. 74) z-scores for Centre I, but -2.01 (-2.19; -1.85) z-score and -3.20 (-3.45; -2.92) z-score respectively for data from Centre F. While theoretically possible, and allowing direct comparison of lung function results to be made using the same equation, such an approach does have practical limitations. Consequently, in an attempt to derive a better model fit, data from Centres E and F were excluded before recalculating the GLI-adjustment for the remaining group (A_1, H & I: Model 3b). See Fig 4, Table 7 and text in main manuscript for details of Model 3b.

2.2 Forced expiratory flows

In keeping with recommendations from the American Thoracic Society/ European Respiratory Society not to report numeric data derived from flow-volume curves[16] and increasing evidence that forced expiratory flows do not offer any interpretative advantage over FEV$_1$/FVC[17-20], we have not derived South Asian prediction equations for forced expiratory flows.

2.3 Proposal for prospective data collection

S8 Table. Data required for prospective data collection

Study information	Essential details
Measures of ethnicity	Ethnic origin of parents and grandparents; place of birth of three generations; genetic ancestry; main language spoken
Birth details	Date of birth, birth weight and gestation where feasible
Medical history	Chronic or current medical conditions; current symptoms
Socio-economic circumstances (SEC)	Measures that have local and international currency at individual (e.g. maternal education) and area level (area deprivation). Preferably several measures of SEC.
Environmental exposures	Tobacco smoke exposure, maternal and household; outdoor and indoor air pollution
Standardised anthropometric assessments	Standing and sitting height, weight
Lung function assessments	Performed according to ATS/ERS guidelines using equipment that allows prospective quality control at time of data collection, storage of all data for subsequent independent over-read and automated export of results to avoid transcription errors.
Recording of age and height	To one decimal place (in years, cm)

3 References

1. Sonnappa S, Lum S, Kirkby J, Bonner R, Wade A, et al. (2015) Disparities in pulmonary function in healthy children across the Indian urban-rural continuum. Am J Respir Crit Care Med 191: 79-86.
2. Chhabra SK, Vijayan VK, Rahman M, Mittal V, Singh PD (2012) Regression equations for spirometry in children aged 6 to 17 years in Delhi region. Indian J Chest Dis Allied Sci 54: 59-63.
3. Doctor TH, Trivedi SS, Chudasama RK (2010) Pulmonary function test in healthy school children of 8 to 14 years age in south Gujarat region, India. Lung India 27: 145-148.
4. Raju PS, Prasad KVV, Ramana YV, Murthy KJ (2004) Pulmonary function tests in Indian girls--prediction equations. Indian J Pediatr 71: 893-897.
5. Raju PS, Prasad KVV, Ramana YV, Ahmed SK, Murthy KJ (2003) Study on lung function tests and prediction equations in Indian male children. Indian Pediatr 40: 705-711.
6. Barone-Adesi F, Dent JE, Dajnak D, Beevers S, Anderson HR, et al. (2015) Long-Term Exposure to Primary Traffic Pollutants and Lung Function in Children: Cross-Sectional Study and Meta-Analysis. PLoS One 10: e0142565.
7. Whitrow MJ, Harding S (2008) Ethnic differences in adolescent lung function: anthropometric, socioeconomic, and psychosocial factors. Am J Respir Crit Care Med 177: 1262-1267.
8. Whittaker AL, Sutton AJ, Beardsmore CS (2005) Are ethnic differences in lung function explained by chest size? Arch Dis Child Fetal Neonatal Ed 90: F423-428.
9. Strippoli MP, Kuehni CE, Dogaru CM, Spycher BD, McNally T, et al. (2013) Etiology of ethnic differences in childhood spirometry. Pediatrics 131: e1842-1849.

10. Lum S, Bountziouka V, Sonnappa S, Wade A, Cole TJ, et al. (2015) Lung function in children in relation to ethnicity, physique and socioeconomic factors. Eur Respir J 46: 1662-1671.
11. ERS (2015) Global lung function initiative: Equation and Tools. ERS e-learning resources: Guidelines.
12. Quanjer PH, Stanojevic S, Cole TJ, Baur X, Hall GL, et al. (2012) Multi-ethnic reference values for spirometry for the 3-95-yr age range: the global lung function 2012 equations. Eur Respir J 40: 1324-1343.
13. Stanojevic S, Quanjer PH, Miller RM, Stocks J (2013) The Global Lung Function Initiative: dispelling some myths of lung function test interpretation. Breathe 9: 463-474.
14. Cleveland WS (1979) Robust locally weighted regression and smoothing scatterplots. J Am Stat Assoc 74: 829-836.
15. Khadilkar VV, Khadilkar AV, Cole TJ, Sayyad MG (2009) Cross-sectional growth curves for height, weight and body mass index for affluent Indian children, 2007. Indian Pediatr 46: 477-489.
16. Pellegrino R, Viegi G, Brusasco V, Crapo RO, Burgos F, et al. (2005) Interpretative strategies for lung function tests. Eur Respir J 26: 948-968.
17. Lukic KZ, Coates AL (2015) Does the FEF25-75 or the FEF75 have any value in assessing lung disease in children with cystic fibrosis or asthma? Pediatr Pulmonol.
18. Boutin B, Koskas M, Guillo H, Maingot L, La Rocca MC, et al. (2015) Forced expiratory flows' contribution to lung function interpretation in schoolchildren. Eur Respir J 45: 107-115.
19. Quanjer PH, Weiner DJ, Pretto JJ, Brazzale DJ, Boros PW (2014) Measurement of FEF25-75% and FEF75% does not contribute to clinical decision making. Eur Respir J 43: 1051-1058.
20. Pellegrino R, Brusasco V, Miller MR (2014) Question everything. Eur Respir J 43: 947-948.
