# Supplementary material for: Challenges in Collating Spirometry Reference Data for South-Asian Children: An Observational Study
Source: PLoS One. 2016 Apr 27;11(4):e0154336. doi: 10.1371/journal.pone.0154336 (PMC4847904; doi:10.1371/journal.pone.0154336)
Supplement: S1 Table — (PDF) [file pone.0154336.s008.pdf]

**S1 Table. Recruitment and exclusion criteria according to respective studies**

| | **Recruitment criteria** | **Exclusion criteria for deriving reference population for this study** |
|---|---|---|
| Bangalore[1] | School children 5 to 12 years of age | Children with overt signs of illness on test day; those with current or chronic respiratory disease or significant congenital abnormalities likely to influence lung function |
| Delhi[2] | School children of North Indian origin, determined by mother tongue & parentage, aged 6 to 17y, screened by a health questionnaire and physical examination. Only “normal” children were assessed. | |
| Gujarat[3] | Studying in class V to VIII aged 8 to 14y during November 2007 to April 2008 | Children with history of (h/o) febrile illness in the last 2 weeks, upper respiratory tract infections like symptoms in the past 2 weeks, acute or chronic respiratory disease, any major systemic disease like cardiac or renal problems, clinical significant anaemia, h/o drug intake which can affect lung function; any allergy; children with bone deformity of chest or spine and any muscular weakness, family h/o atopy, asthma or other chronic lung diseases. |
| Hyderabad[4,5] | Healthy children aged between 5 and 15y | Children with any respiratory disease or had recent history of respiratory infections. |
| CHASE[6]* | Primary school children aged 9 to 10y | Gestational age <37 w; Children with current or chronic respiratory disease or significant congenital abnormalities likely to influence lung function. |
| DASH[7]* | Children from Year 7 and 8 (11-13 years old) | Gestational age <37 w; current or chronic respiratory disease or significant congenital abnormalities likely to influence lung function; |
| Leicester city[8]* | Children aged 6-11 years from nine city primary schools | Children with a BMI >30kg/m$^2$, h/o cardio-pulmonary disease, chest wall deformity, or preterm delivery. Although Asthma was not an exclusion criterion unless the child required daily medication, children with a diagnosis of asthma were not included in the collated dataset. |
| Leicester Respiratory Cohort[9]* | | Gestational age <37 w; Children with current or chronic respiratory disease or significant congenital abnormalities likely to influence lung function. |
| SLIC[10]* | School children between 5 and 12 years of age | Gestational age <37 w; Children with current or chronic respiratory disease or significant congenital abnormalities likely to influence lung function. |

*Studies where recruitment criteria were broader due to their specific study aims but authors were requested to only submit data from healthy children (see exclusion criteria).

For reference list, please see S1 File for details
