# Supplementary material for: Challenges in Collating Spirometry Reference Data for South-Asian Children: An Observational Study
Source: PLoS One. 2016 Apr 27;11(4):e0154336. doi: 10.1371/journal.pone.0154336 (PMC4847904; doi:10.1371/journal.pone.0154336)
Supplement: S2 Table — (PDF) [file pone.0154336.s009.pdf]

**S2 Table. Comparison of anthropometry between children residing in the UK and in India**

| | UK | India | Mean(95%CI) difference (UK-India) |
|---|---|---|---|
| N (% boys) | 3484 (52.1%) | 4640 (59.6%) | -7% (-10%; -5%)*** |
| Age (y) | 10.6 (1.7) | 10.3 (2.9) | 0.3 (0.2; 0.4)*** |
| zHeight | 0.20 (1.02) | -0.36 (1.14) | 0.56 (0.51; 0.61)*** |
| zWeight | 0.24 (1.03) | -0.60 (1.06) | 0.84 (0.80; 0.89)*** |

Data presented as Mean (SD) unless otherwise specified. *** p< 0.0001; Height and weight were expressed as z-scores according to the Indian reference standard, which was based on well-nourished children.[15]
