# Supplementary material for: Challenges in Collating Spirometry Reference Data for South-Asian Children: An Observational Study
Source: PLoS One. 2016 Apr 27;11(4):e0154336. doi: 10.1371/journal.pone.0154336 (PMC4847904; doi:10.1371/journal.pone.0154336)
Supplement: S3 Table — (PDF) [file pone.0154336.s010.pdf]

S3 Table. Preliminary GLI-adjustments according to the various models

	FEV_1		FVC		FEV_1/FVC	
Centres	M	S	M	S	M	S
Model 1 (B)	-0.0853	0.1056	-0.0690	0.0802	-0.0210	-0.0344
Model 2 (A_{2-3}, C)	-0.2108	0.1056	-0.2089	0.0802	0.0032	-0.0344
Model 3a (A_1,E,F,H,I)	-0.1518	0.1056	-0.1432	0.0802	-0.0147	-0.0344
Model 3b (A_1,H,I)	-0.1294	0.1056	-0.1224	0.0802	-0.0135	-0.0344

Abbreviations: M=**M**u (median) or predicted value; S=**S**igma (coefficient of variation), which models the spread of values around the median and adjusts for any non-uniform dispersion.
Centres: A_1=Bangalore, urban; A_{2-3}=Bangalore, semi-urban & rural; B=Delhi; C=Gujarat; E=CHASE; F=DASH; H=Leicester Respiratory Cohort; I=SLIC; Model 3b: final/definitive model. The values of M indicate that when compared with the GLI reference for White subjects (calculated as 100*(1-exp (M)), FEV_1 and FVC were on average ~7% lower for Model 1; ~19% lower for Model 2 and ~12% lower for Model 3b with a relatively constant FEV_1/FVC across the models (Model 1: 2%; Model 2: 0.3%; Model 3a: 1.5%; Model 3b: 1.3%).
See above S1 File section 1.2.1 for details on how to apply these preliminary GLI-adjustments.
