# Supplementary material for: Challenges in Collating Spirometry Reference Data for South-Asian Children: An Observational Study
Source: PLoS One. 2016 Apr 27;11(4):e0154336. doi: 10.1371/journal.pone.0154336 (PMC4847904; doi:10.1371/journal.pone.0154336)
Supplement: S4 Table — (PDF) [file pone.0154336.s011.pdf]

S4 Table. Lung function results based on Model 1 GLI-coefficients derived from Centre B (Delhi)

Centre	n	$zFEV_1$	zFVC	$zFEV_1/FVC$	% ≤LLN $zFEV_1$	% ≤LLN zFVC	% ≤LLN $zFEV_1/FVC$
B	670	0.02 (0.86)	0.02 (0.89)	0.09 (1.06)	2.1%	2.2%	4.2%

Data presented as Mean (SD) unless otherwise specified. Abbreviations: LLN: Lower limit of normal (equates to ≤ -1.645 z-scores)
