# Supplementary material for: Challenges in Collating Spirometry Reference Data for South-Asian Children: An Observational Study
Source: PLoS One. 2016 Apr 27;11(4):e0154336. doi: 10.1371/journal.pone.0154336 (PMC4847904; doi:10.1371/journal.pone.0154336)
Supplement: S5 Table — (PDF) [file pone.0154336.s012.pdf]

S5 Table. Lung function results based on Model 2 GLI-coefficients derived from Centres A_{2-3} & C

Centre	n	$zFEV_1$	zFVC	$zFEV_1/FVC$	% ≤LLN $zFEV_1$	% ≤LLN zFVC	% ≤LLN $zFEV_1/FVC$
A_{2-3}	399	0.03(0.94)	0.11(1.01)	-0.18(0.93)	3.3%	4.3%	3.3%
C	648	0.07(1.02)	-0.03(1.09)	0.10(0.89)	3.4%	4.6%	3.1%
Total	1047	0.05(0.99)	0.02(1.06)	-0.01(0.91)	3.3%	4.5%	3.2%

Data presented as Mean (SD) unless otherwise specified. Abbreviations: LLN: Lower limit of normal (i.e. 5th centile which equates to ≤ -1.645 z-scores). Centre A_{2-3}: Bangalore (semi-urban & rural); Centre C: Gujarat
