# Supplementary material for: Challenges in Collating Spirometry Reference Data for South-Asian Children: An Observational Study
Source: PLoS One. 2016 Apr 27;11(4):e0154336. doi: 10.1371/journal.pone.0154336 (PMC4847904; doi:10.1371/journal.pone.0154336)
Supplement: S6 Table — (PDF) [file pone.0154336.s013.pdf]

S6 Table. Lung function results based on GLI-coefficients derived from Centres A_1(urban), E, F, H & I (Model 3a)

Centres	N	$zFEV_1$	zFVC	$zFEV_1$/FVC	%≤LLN $zFEV_1$	%≤LLN zFVC	% ≤LLN $zFEV_1$/FVC	Adj LLN‡ $zFEV_1$	Adj LLN‡ zFVC	Adj LLN‡ $zFEV_1$/FVC
A_1	383	0.27(0.90)	0.27(0.90)	0.06(0.88)	1.8%	1.6%	2.9%	-1.20	-1.24	-1.39
E	1547	0.07(1.26)	0.02(1.30)	0.31(1.40)	6.2%	6.9%	8.3%	-1.79	-1.84	-2.06
F	1064	-0.01(1.11)	0.08(1.69)	0.37(1.77)	7.0%	8.6%	13.4%	-1.83	-2.01	-3.20
H	210	0.09(1.11)	-0.14(1.04)	0.56(1.14)	4.3%	7.6%	3.8%	-1.63	-1.91	-1.31
I	486	0.28(0.92)	0.31(0.90)	0.01(1.01)	1.6%	0.8%	5.6%	-1.21	-1.10	-1.72
Total	3690	0.10(1.14)	0.09(1.34)	0.28(1.42)	5.3%	6.1%	8.6%	-1.67	-1.77	-2.20

Data presented as Mean (SD) unless otherwise specified. Abbreviations: LLN: Lower limit of normal (equates to ≤-1.645 z-scores); Adj LLN‡: LLN adjusted for the actual 5th centile according to each centre. Centres: A_1= Bangalore (urban); E=CHASE; F=DASH; H=Leicester Respiratory Cohort; I= SLIC
