# Supplementary material for: Challenges in Collating Spirometry Reference Data for South-Asian Children: An Observational Study
Source: PLoS One. 2016 Apr 27;11(4):e0154336. doi: 10.1371/journal.pone.0154336 (PMC4847904; doi:10.1371/journal.pone.0154336)
Supplement: S7 Table — (PDF) [file pone.0154336.s014.pdf]

S7 Table. Lung function results based on Model 3b GLI-coefficients derived from Centres A_1, H and I

Centre	n	$zFEV_1$	zFVC	$zFEV_1/FVC$	%≤ LLN $zFEV_1$	%≤LLN zFVC	% ≤ LLN $zFEV_1/FVC$
A_1	383	0.09(0.88)	0.11(0.88)	0.05(0.87)	2. 3%	2.6%	2.9%
H	210	-0.09(1.08)	-0.30(1.02)	0.54(1.13)	5.2%	9.0%	3.8%
I	486	0.10(0.89)	0.15(0.88)	-0.01(1.00)	2.5%	1.2%	5.6%
Total	1079	0.06(0.93)	0.05(0.92)	0.12(1.01)	3.0%	3.2%	4.3%

Data presented as Mean (SD) unless otherwise specified. Abbreviations: LLN: Lower limit of normal (equates to ≤ -1.645 z-scores). Centre A_1: Bangalore (urban); Centre H: Leicester Respiratory Cohort; Centre I: SLIC
