# Supplementary material for: Challenges in Collating Spirometry Reference Data for South-Asian Children: An Observational Study
Source: PLoS One. 2016 Apr 27;11(4):e0154336. doi: 10.1371/journal.pone.0154336 (PMC4847904; doi:10.1371/journal.pone.0154336)
Supplement: S8 Table — (PDF) [file pone.0154336.s015.pdf]

S8 Table. Data required for prospective data collection

Study information	Essential details
Measures of ethnicity	Ethnic origin of parents and grandparents; place of birth of three generations; genetic ancestry; main language spoken
Birth details	Date of birth, birth weight and gestation where feasible
Medical history	Chronic or current medical conditions; current symptoms
Socio-economic circumstances (SEC)	Measures that have local and international currency at individual (e.g. maternal education) and area level (area deprivation). Preferably several measures of SEC.
Environmental exposures	Tobacco smoke exposure, maternal and household; outdoor and indoor air pollution
Standardised anthropometric assessments	Standing and sitting height, weight
Lung function assessments	Performed according to ATS/ERS guidelines using equipment that allows prospective quality control at time of data collection, storage of all data for subsequent independent over-read and automated export of results to avoid transcription errors.
Recording of age and height	To one decimal place (in years, cm)
